# Supplementary material for: A patterned human primitive heart organoid model generated by pluripotent stem cell self-organization
Source: Nat Commun. 2023 Dec 12;14:8245. doi: 10.1038/s41467-023-43999-1 (PMC10716495; doi:10.1038/s41467-023-43999-1)
Supplement: Supplementary file 1 — Supplementary Information [file 41467_2023_43999_MOESM1_ESM.pdf]

SUPPLEMENTARY INFORMATION

A patterned human primitive heart organoid model generated by pluripotent stem cell self-organization

Brett Volmert[1,2], Artem Kiselev[1,3,4], Aniwat Juhong[5,6], Fei Wang[7], Ashlin Riggs[1,2], Aleksandra Kostina[1,2], Colin O'Hern[1,2], Priyadharshni Muniyandi[1,2], Aaron Wasserman[1,2], Amanda Huang[1,2], Yonatan Lewis-Israeli[1,2], Vishal Panda[3,8], Sudin Bhattacharya[3,8], Adam Lauver[3], Sangbum Park[1,3,4], Zhen Qiu[5,6], Chao Zhou[7], Aitor Aguirre[1,2,*]

[1]Institute for Quantitative Health Science and Engineering, Division of Developmental and Stem Cell Biology, Michigan State University, MI, USA

[2]Department of Biomedical Engineering, College of Engineering, Michigan State University, MI, USA

[3]Department of Pharmacology and Toxicology, College of Human Medicine, Michigan State University, MI, USA

[4]Division of Dermatology, Department of Medicine, College of Human Medicine, Michigan State University, East Lansing, MI 48824, USA

[5]Institute for Quantitative Health Science and Engineering, Division of Biomedical Devices, Michigan State University, MI, USA

[6]Department of Electrical and Computer Engineering, College of Engineering, Michigan State University, MI, USA

[7]Department of Biomedical Engineering, Washington University in Saint Louis, MO, USA

[8]Institute for Quantitative Health Science and Engineering, Division of Systems Biology, Michigan State University, MI, USA

Correspondence should be addressed to A.A. (email: aaguirre@msu.edu).

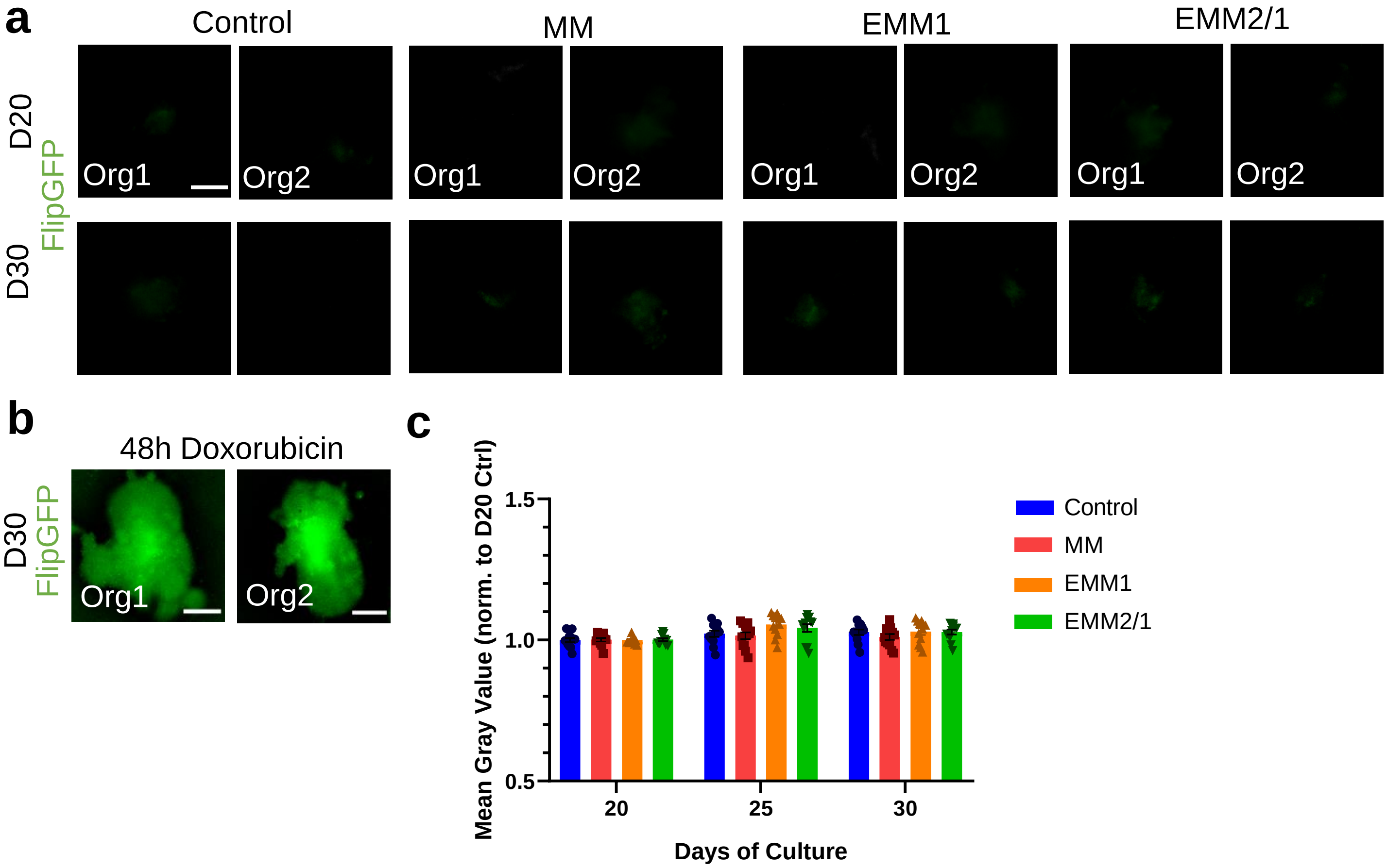

Supplementary Fig. 1. Longitudinal assessment of apoptosis across all maturation conditions. a, Representative fluorescence images of day 20 and day 30 organoids from each condition displaying FlipGFP fluorescence signal (n=12 independent organoids per condition per day across two independent experiments). **b,** Representative fluorescence images of day 30 EMM2/1 organoids following a 48-hour exposure to doxorubicin displaying FlipGFP fluorescence signal (n=6 independent organoids per condition across two independent experiments). **c,** Quantification of fluorescence intensity from images presented in **(a)** (n=12 independent organoids per condition per day across two independent experiments). Data presented as fold change normalized to day 20. Values = mean ± s.e.m., matched two-way ANOVA with Tukey's multiple comparisons test. Source data are provided as a Source Data file.

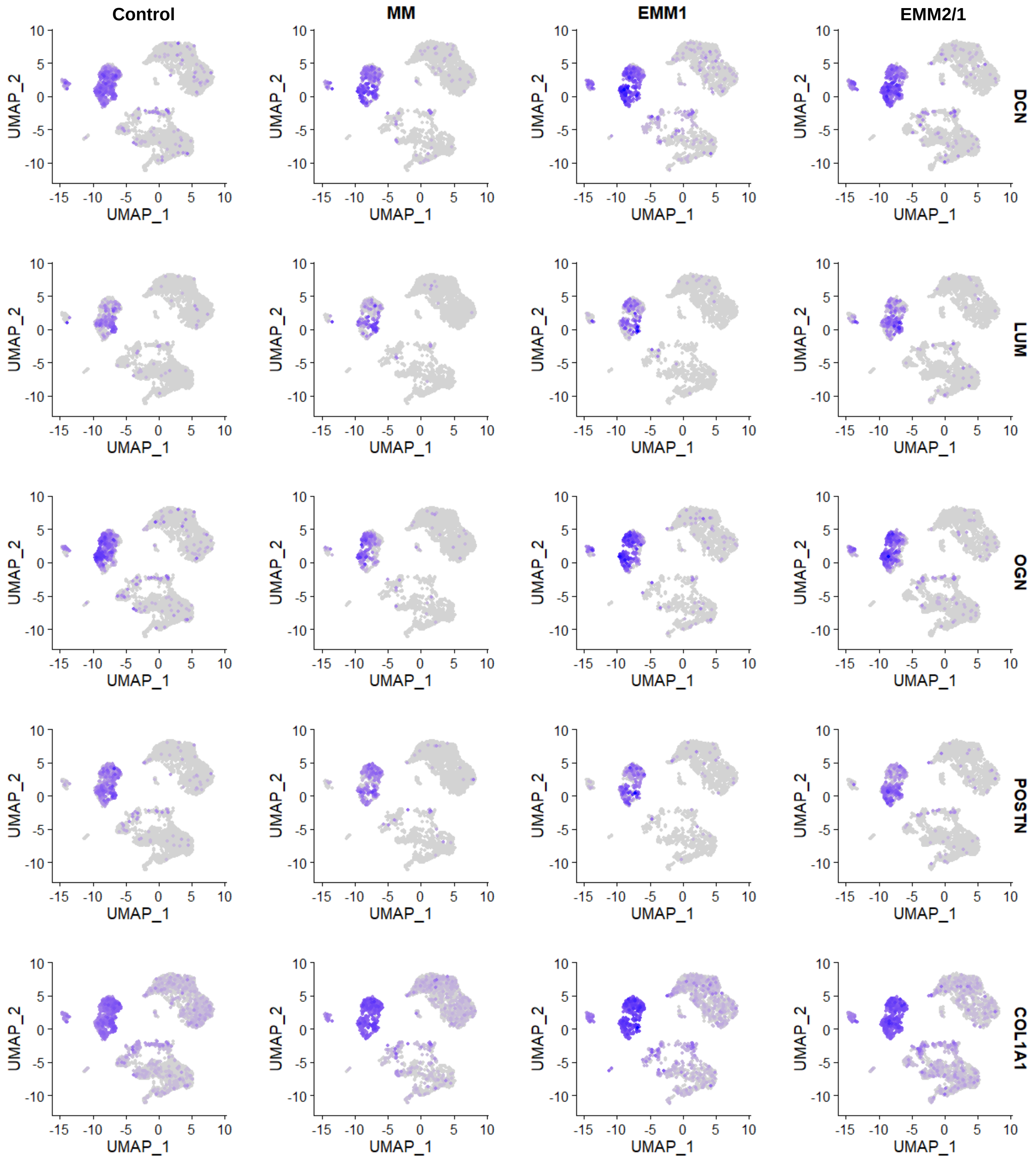

Supplementary Fig. 2. Feature plots of cardiac fibroblast-related genes within single cell RNA sequencing datasets in each condition.

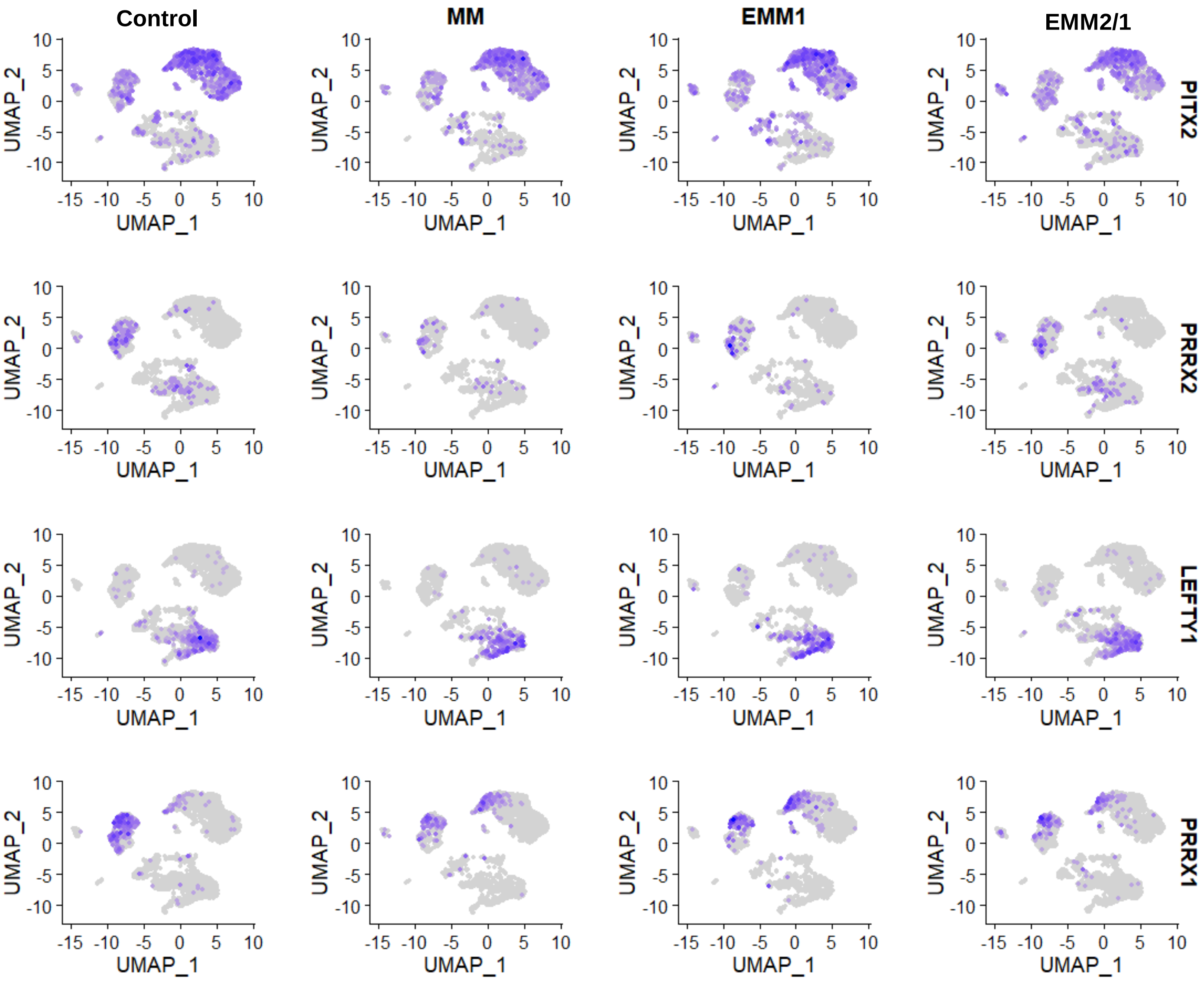

Supplementary Fig. 3. Feature plots of left-right asymmetry-related genes within single cell RNA sequencing datasets in each condition.

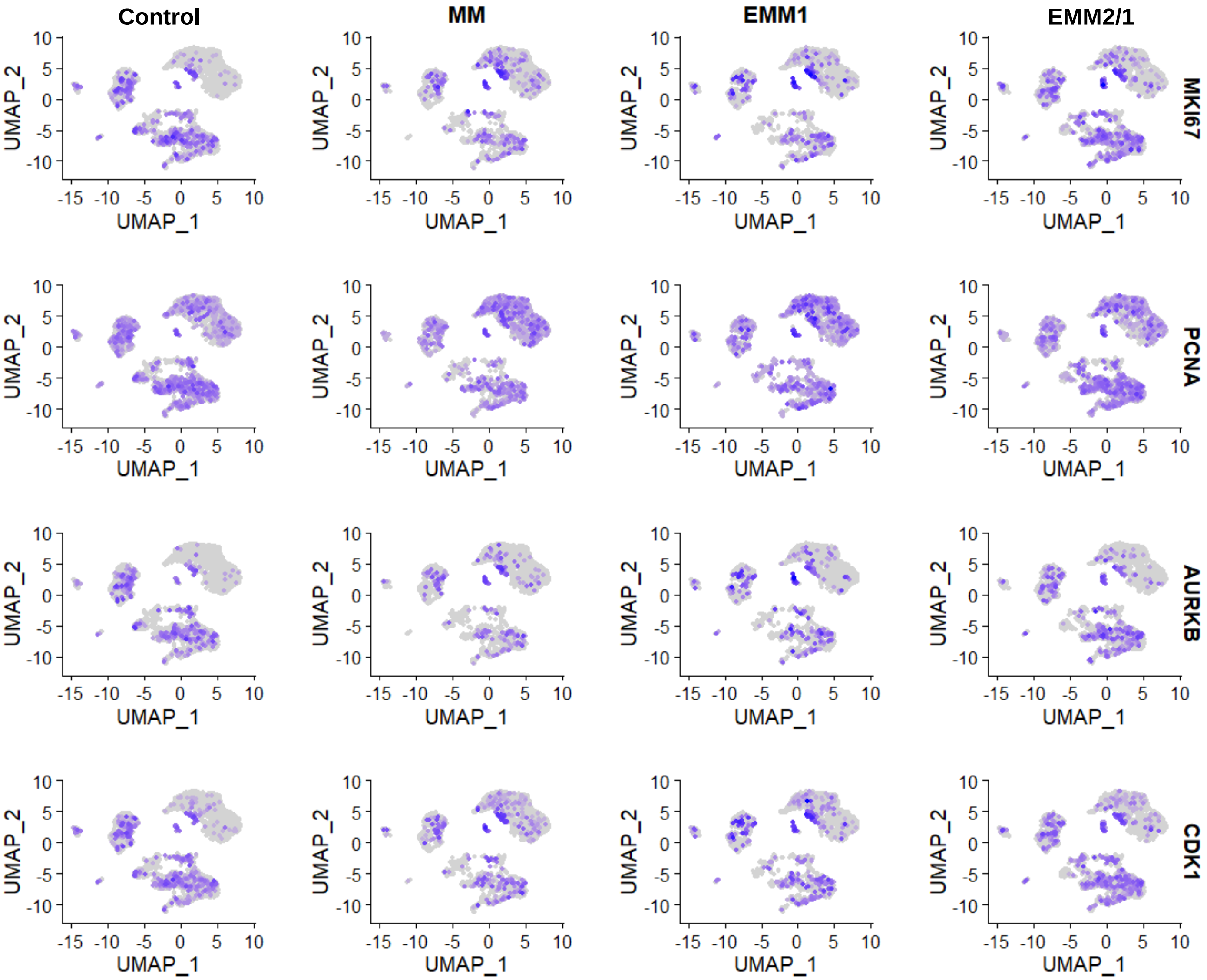

Supplementary Fig. 4. Feature plots of proliferation-related genes within single cell RNA sequencing datasets in each condition.

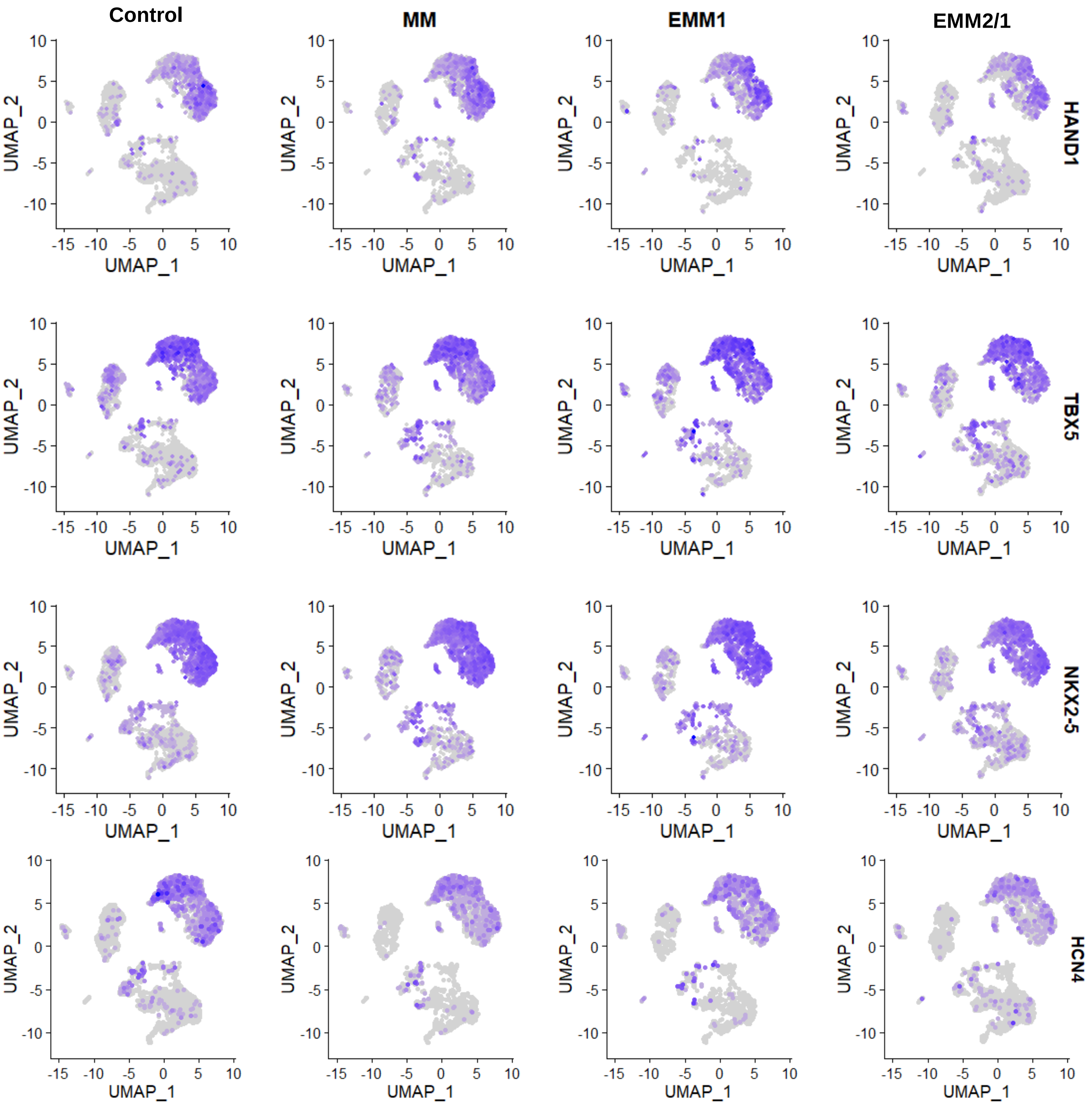

Supplementary Fig. 5. Feature plots of first heart field-related genes within single cell RNA sequencing datasets in each condition.

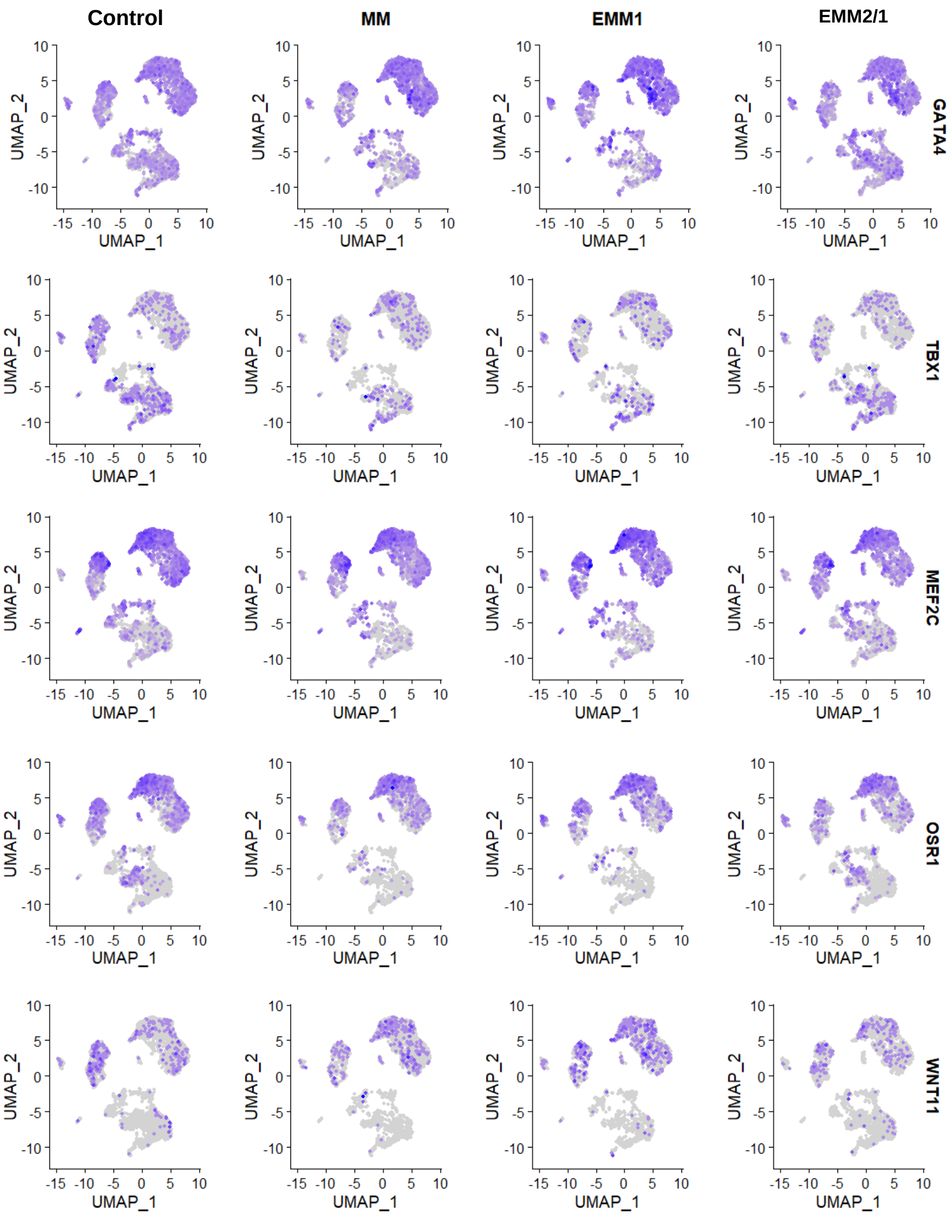

Supplementary Fig. 6. Feature plots of second heart field-related genes within single cell RNA sequencing datasets in each condition.

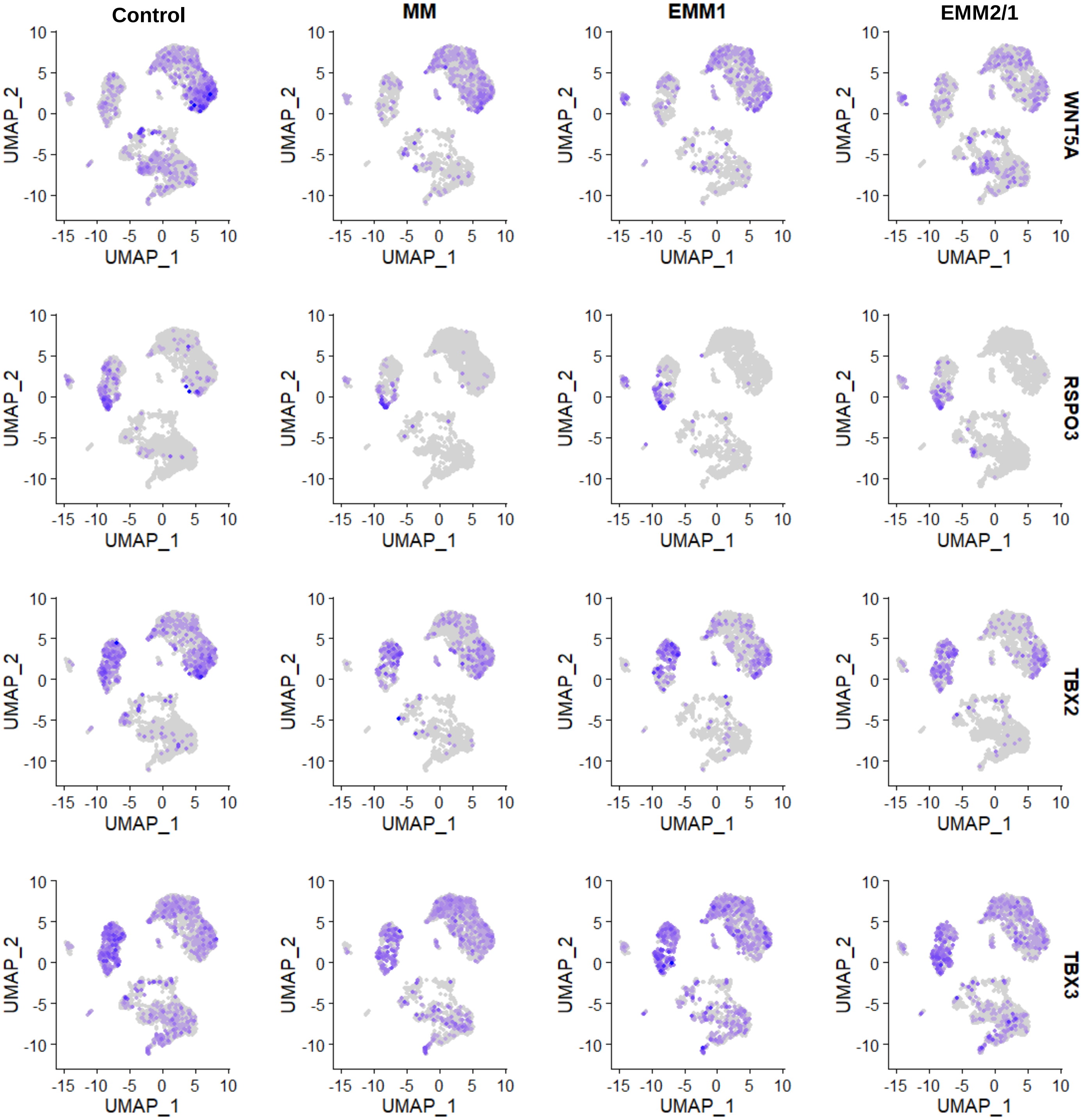

Supplementary Fig. 7. Feature plots of outflow tract-related genes within single cell RNA sequencing datasets in each condition.

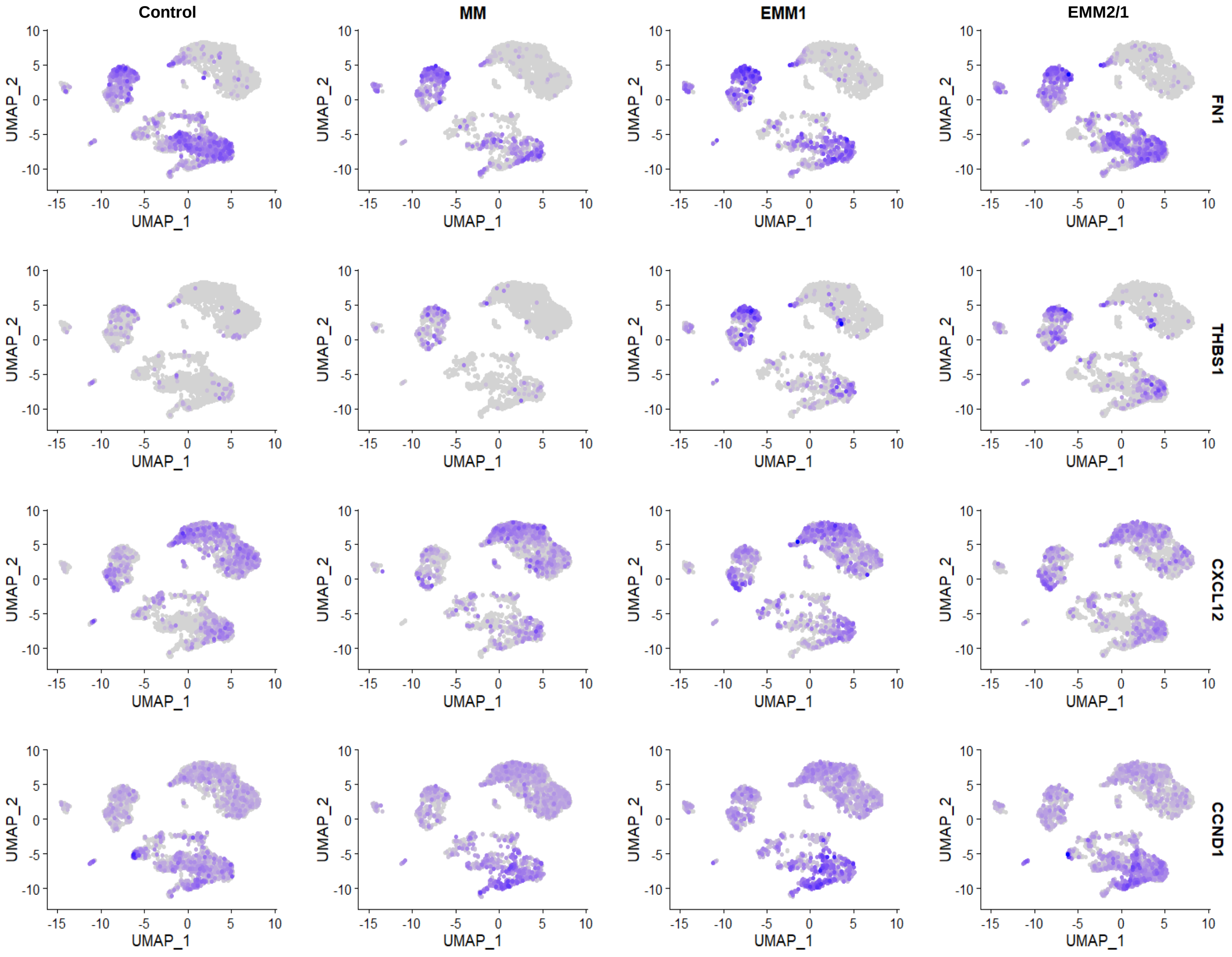

Supplementary Fig. 8. Feature plots of valve development-related genes within single cell RNA sequencing datasets in each condition.

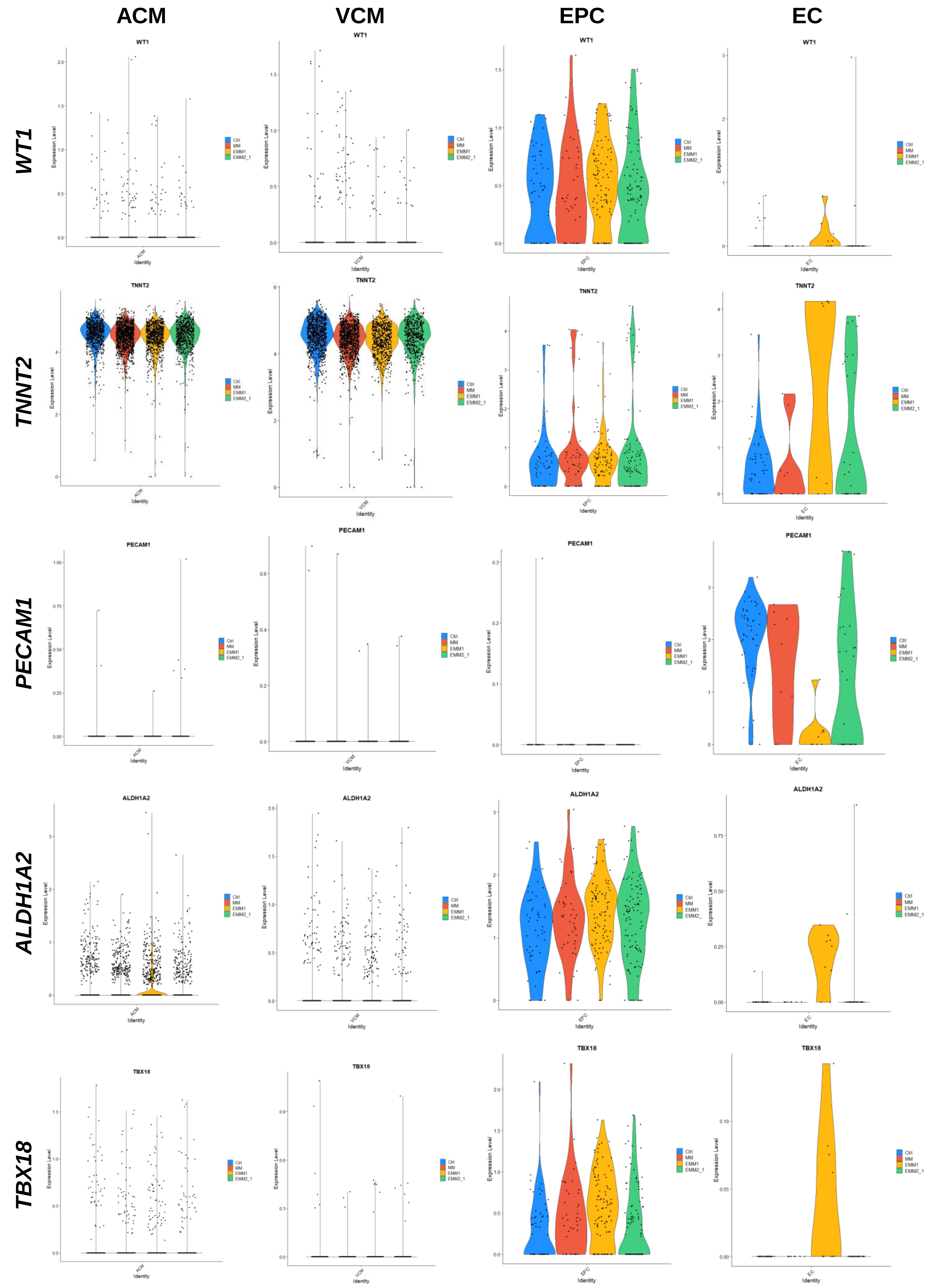

Supplementary Fig. 9. Violin plots for genes in the ACM, VCM, EPC, and EC clusters for all four conditions (control = blue, MM = red, EMM1 = orange, EMM2/1 = green.

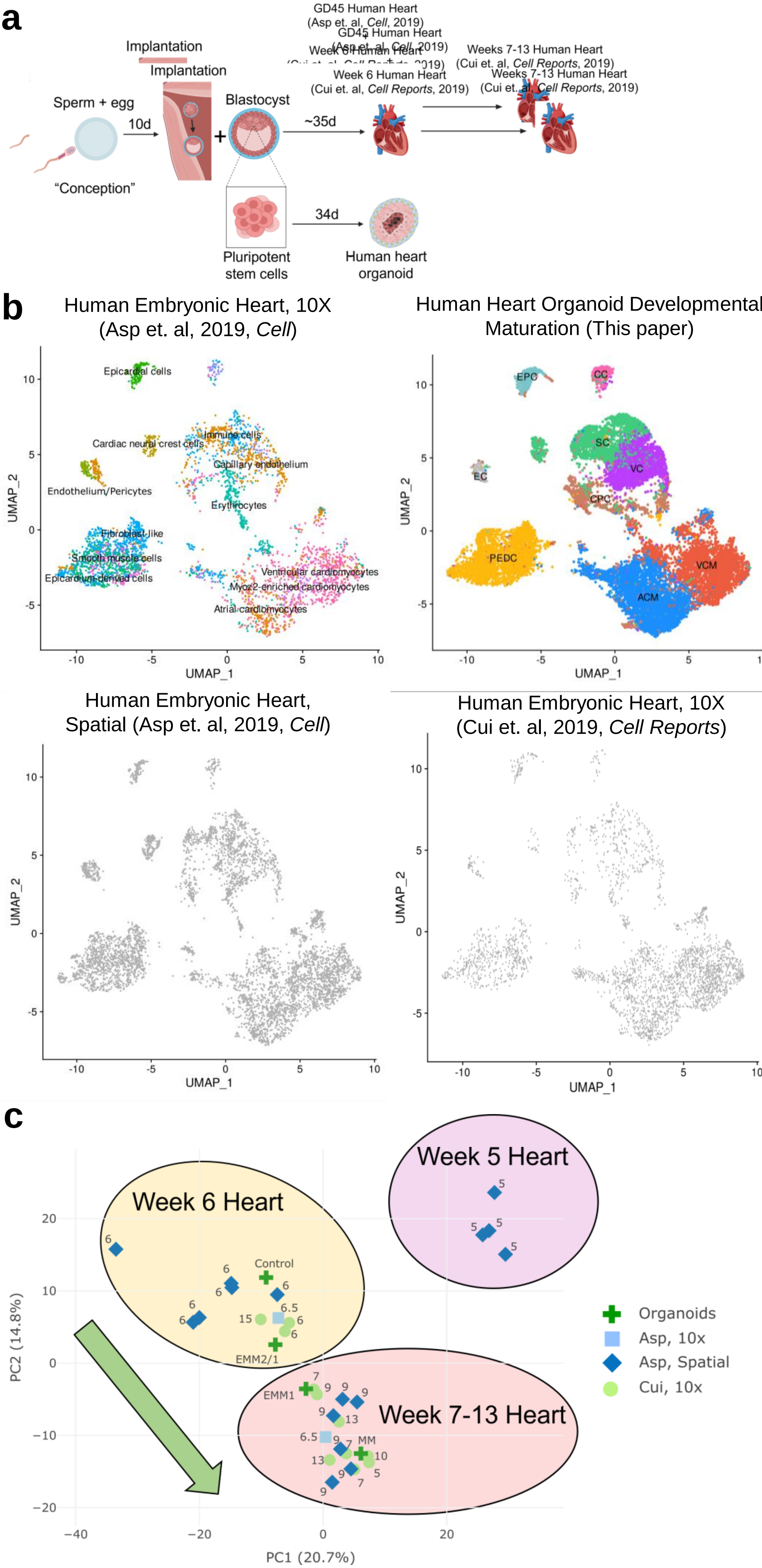

Supplementary Fig. 10

Supplementary Fig. 10. Transcriptomic organoid landscape reveals similarities to *in vivo* developing human hearts. a, Schematic of timeline comparing human embryonic heart development and human heart organoid development. Created using BioRender.com. **b,** UMAP projections displaying human embryonic heart and human heart organoid scRNAseq datasets. Cluster naming for (Asp et al, *Cell*, 2019) is preserved from original text. Cluster identity and color for the human heart organoid dataset is preserved from that shown in **(Fig. 2a)**. **c,** PCA plot for datasets presented in **(b)**.

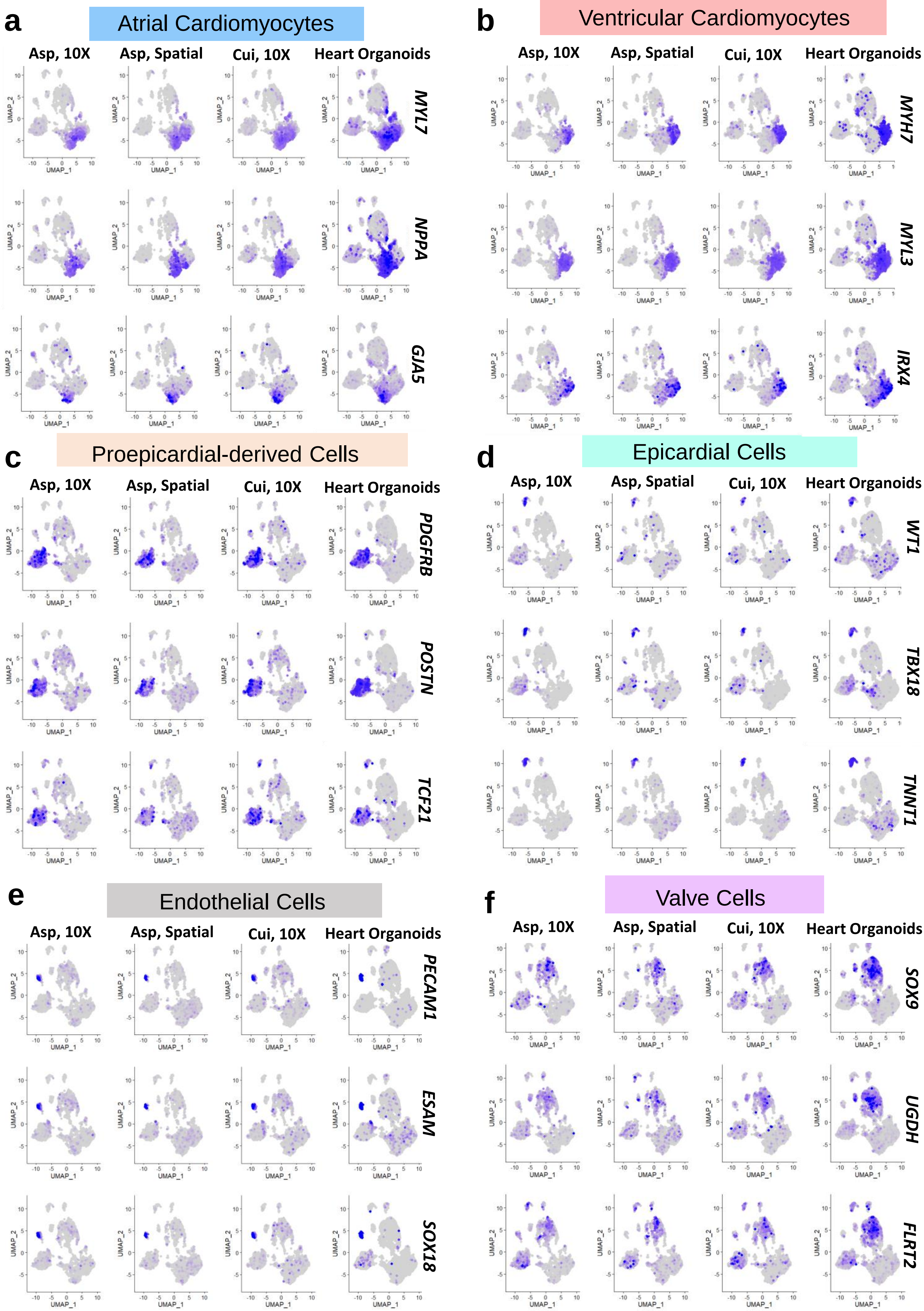

Supplementary Fig. 11

Supplementary Fig. 11. Human heart organoids share key gene expression with embryonic human hearts across cardiac cell types. a-f, Feature plots displaying key marker genes for each cluster in the Asp 2019, Cui 2019, and human heart organoid datasets. Clusters of interest include: Atrial Cardiomyocytes **(a)**; Ventricular Cardiomyocytes **(b)**; Proepicardial-derived Cells **(c)**; Epicardial Cells **(d)**; Endothelial Cells **(e)**; and Valve Cells **(f)**. Color intensity represents the relative value of gene expression per gene.

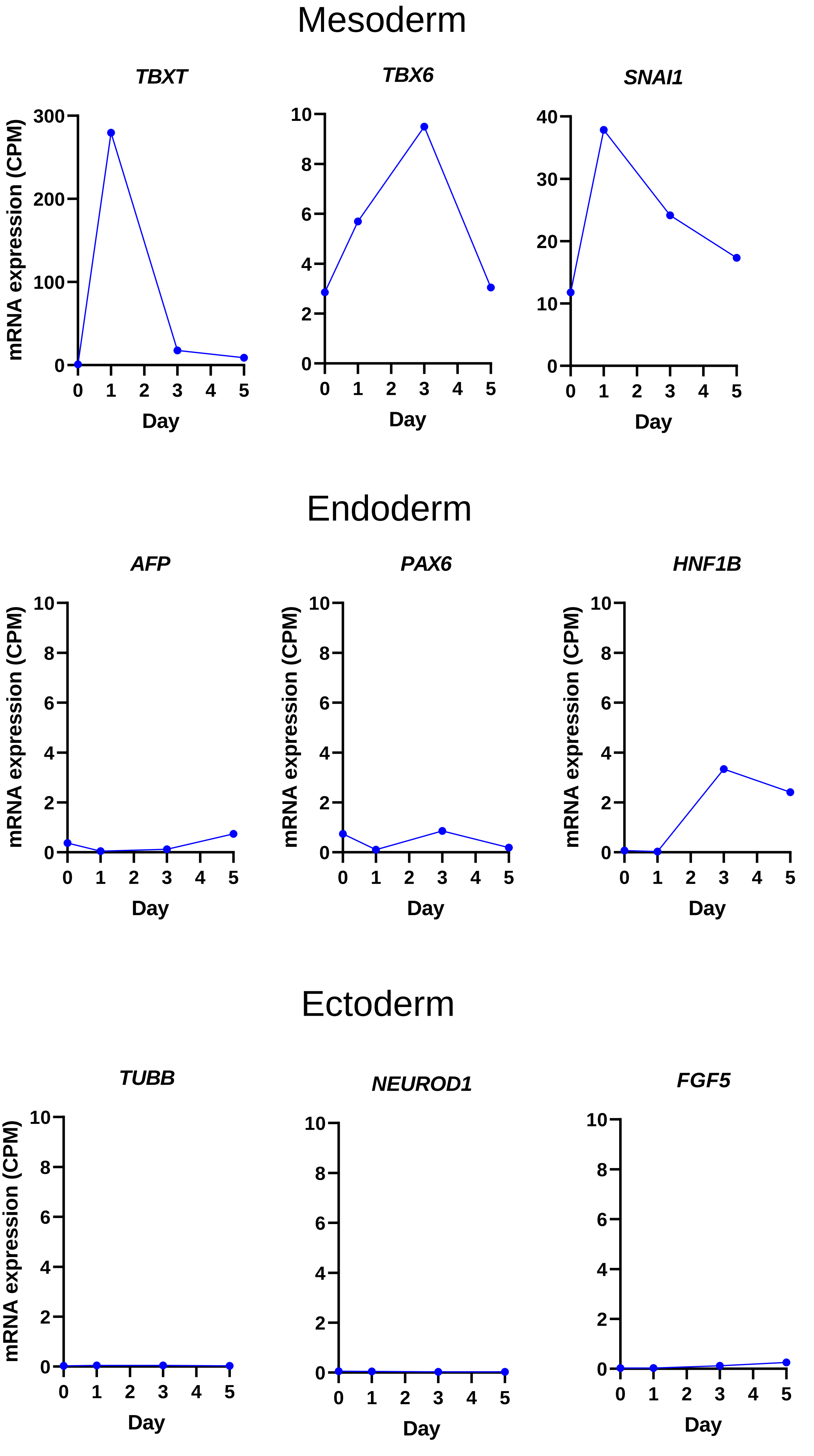

Supplementary Fig. 12. RNA sequencing data from days 0-5 organoid differentiation showing expression values for key mesodermal, endodermal, and ectodermal markers (n=8 organoids per time point). CPM = counts per million. Source data are provided as a Source Data file.

Supplementary Fig. 13. Feature plots of stromal cell **(a)** conductance cell **(b)** and metabolic markers **(c)** across embryonic heart datasets and heart organoid datasets.

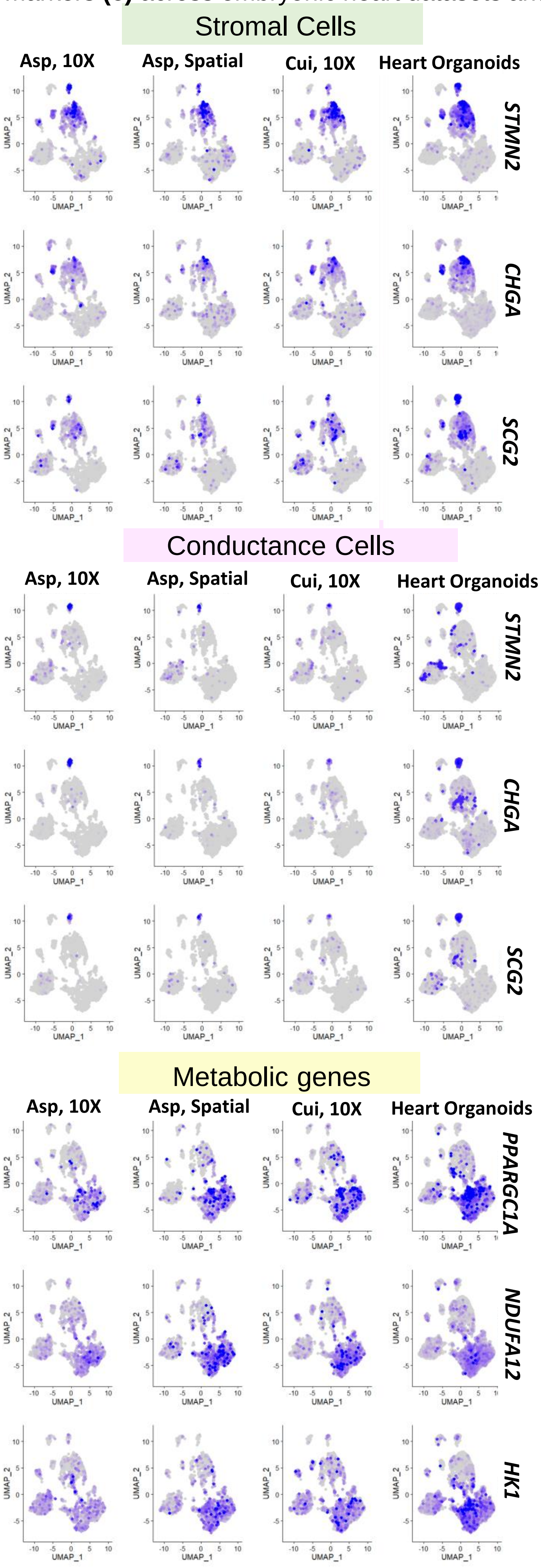

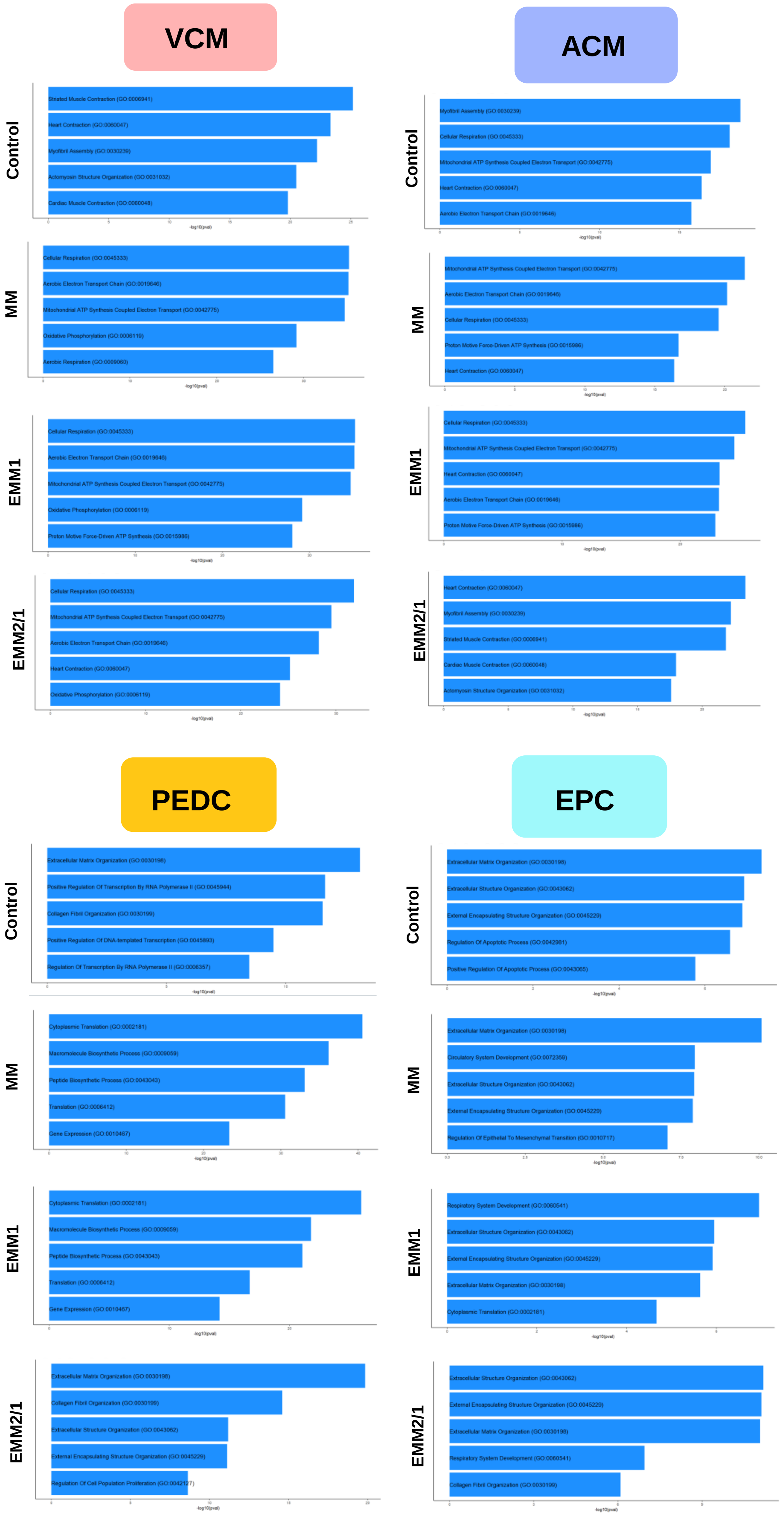

Supplementary Fig. 14. Top 5 positively regulated GO Biological Processes using the top 250 differentially expressed genes within the VCM, ACM, PEDC and EPC clusters for each condition.

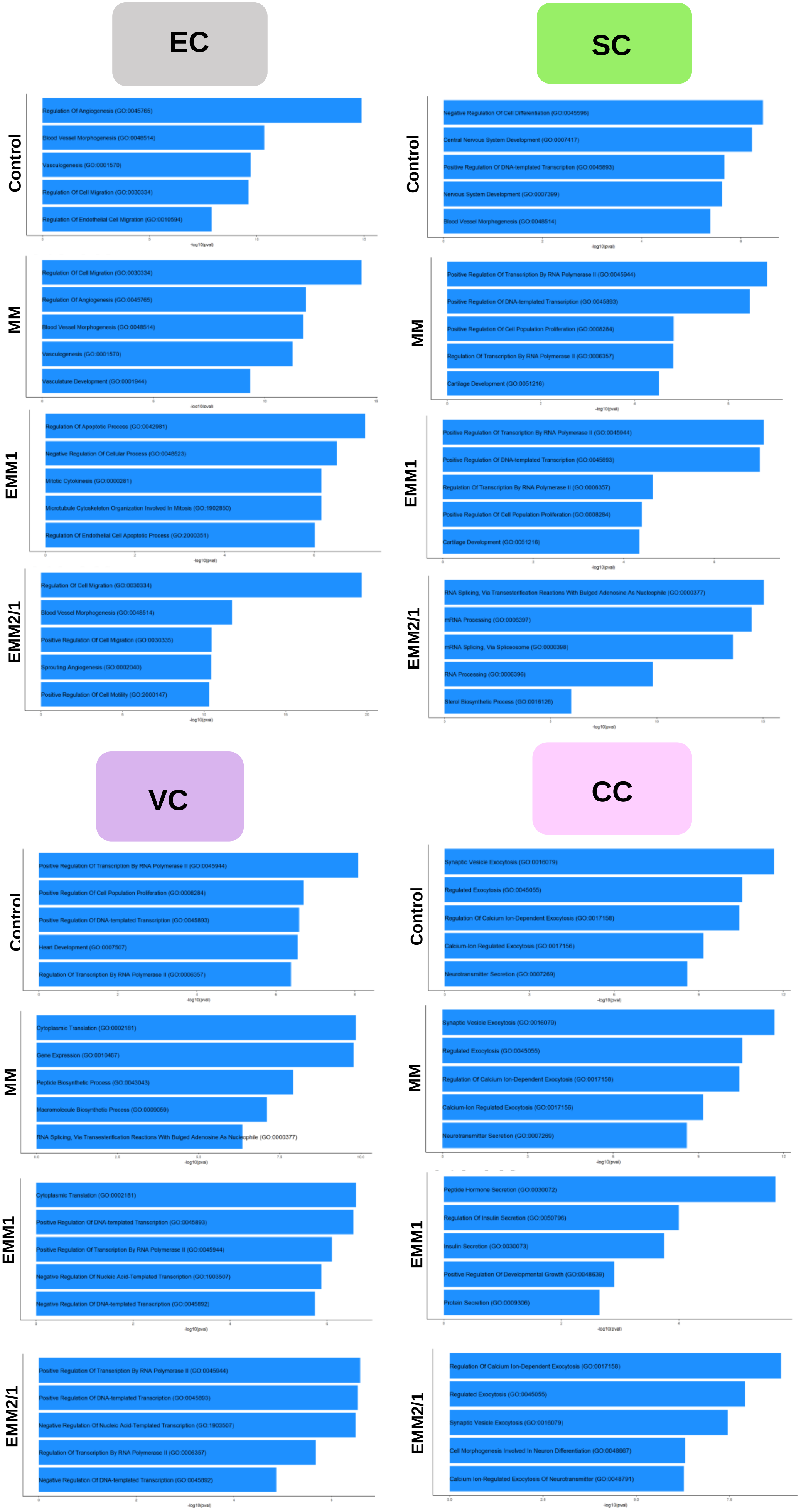

Supplementary Fig. 15. Top 5 positively regulated GO Biological Processes using the top 250 differentially expressed genes within the EC, SC, VC, and CC clusters for each condition.

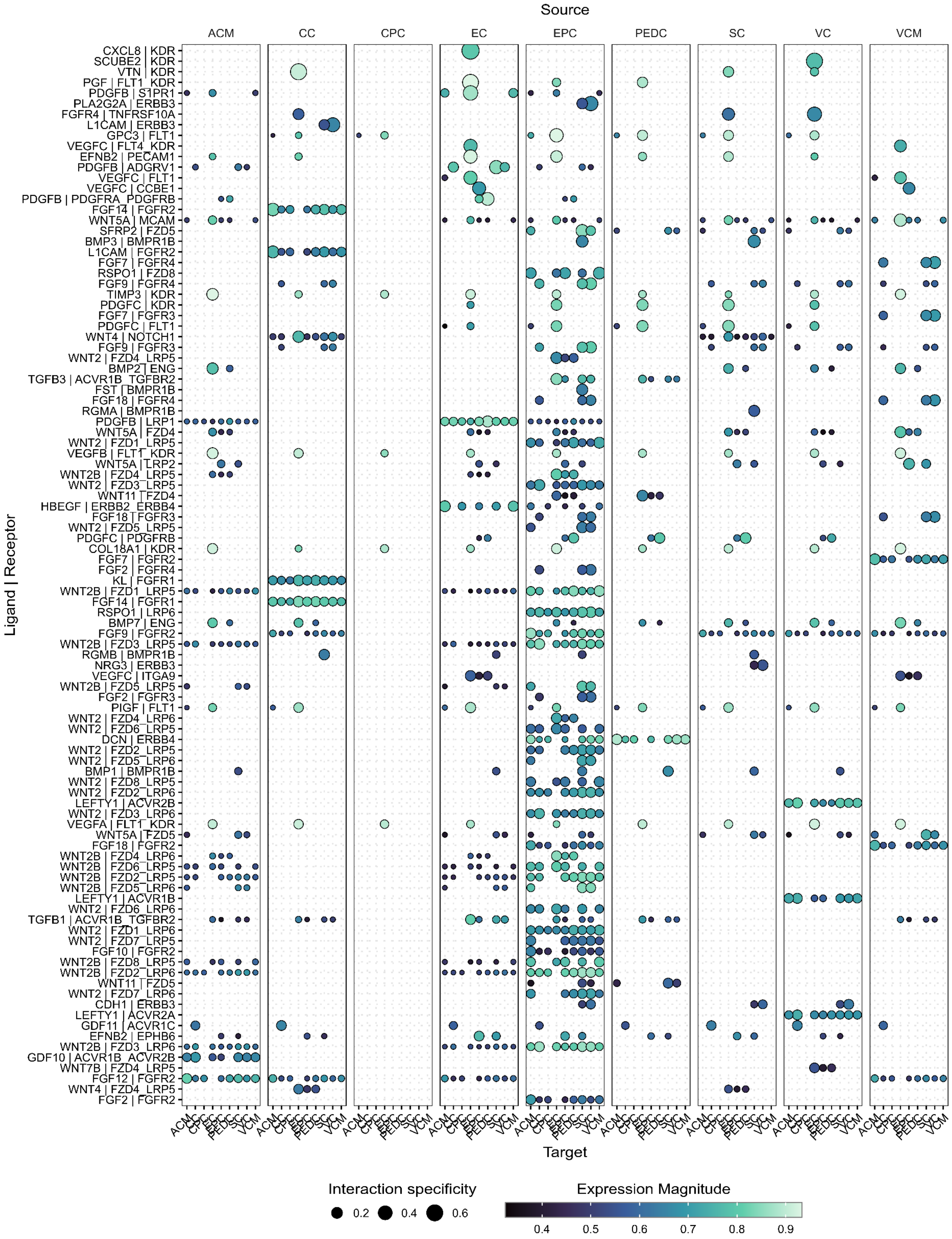

Supplementary Fig. 16. Cell-cell communication via ligand-receptor pairing for each cluster using scRNAseq data from the control condition.

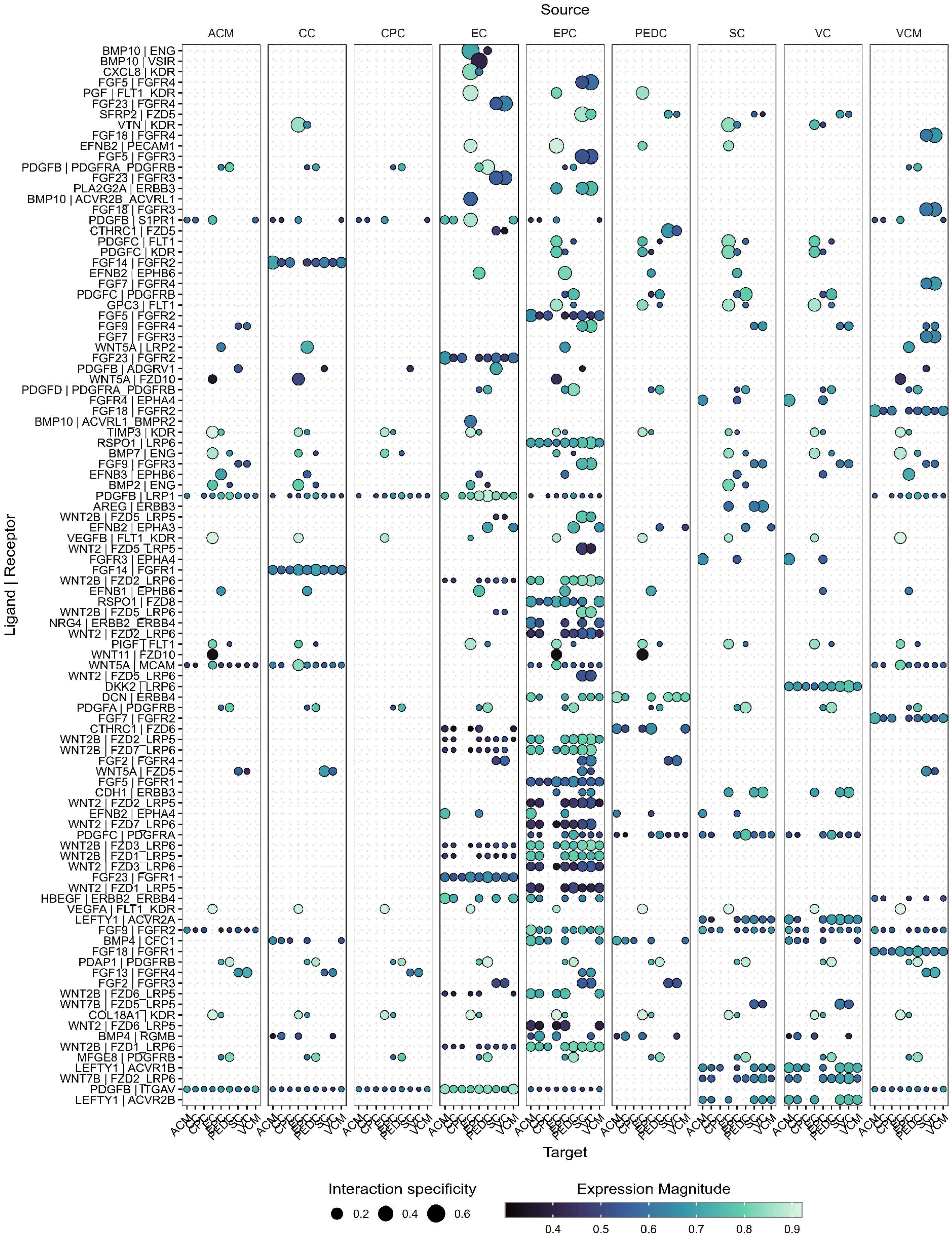

Supplementary Fig. 17. Cell-cell communication via ligand-receptor pairing for each cluster using scRNAseq data from the MM condition.

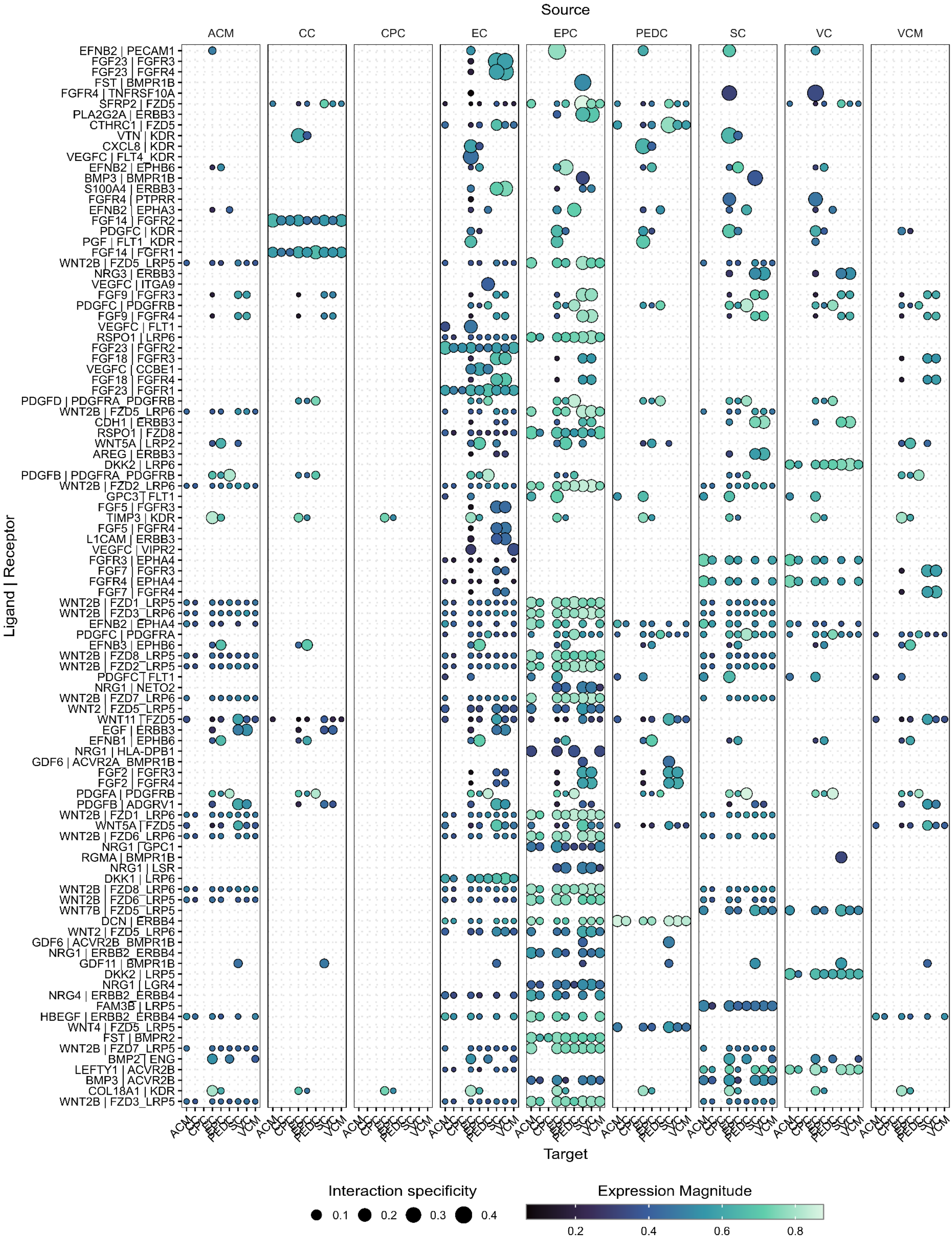

Supplementary Fig. 18. Cell-cell communication via ligand-receptor pairing for each cluster using scRNAseq data from the EMM1 condition.

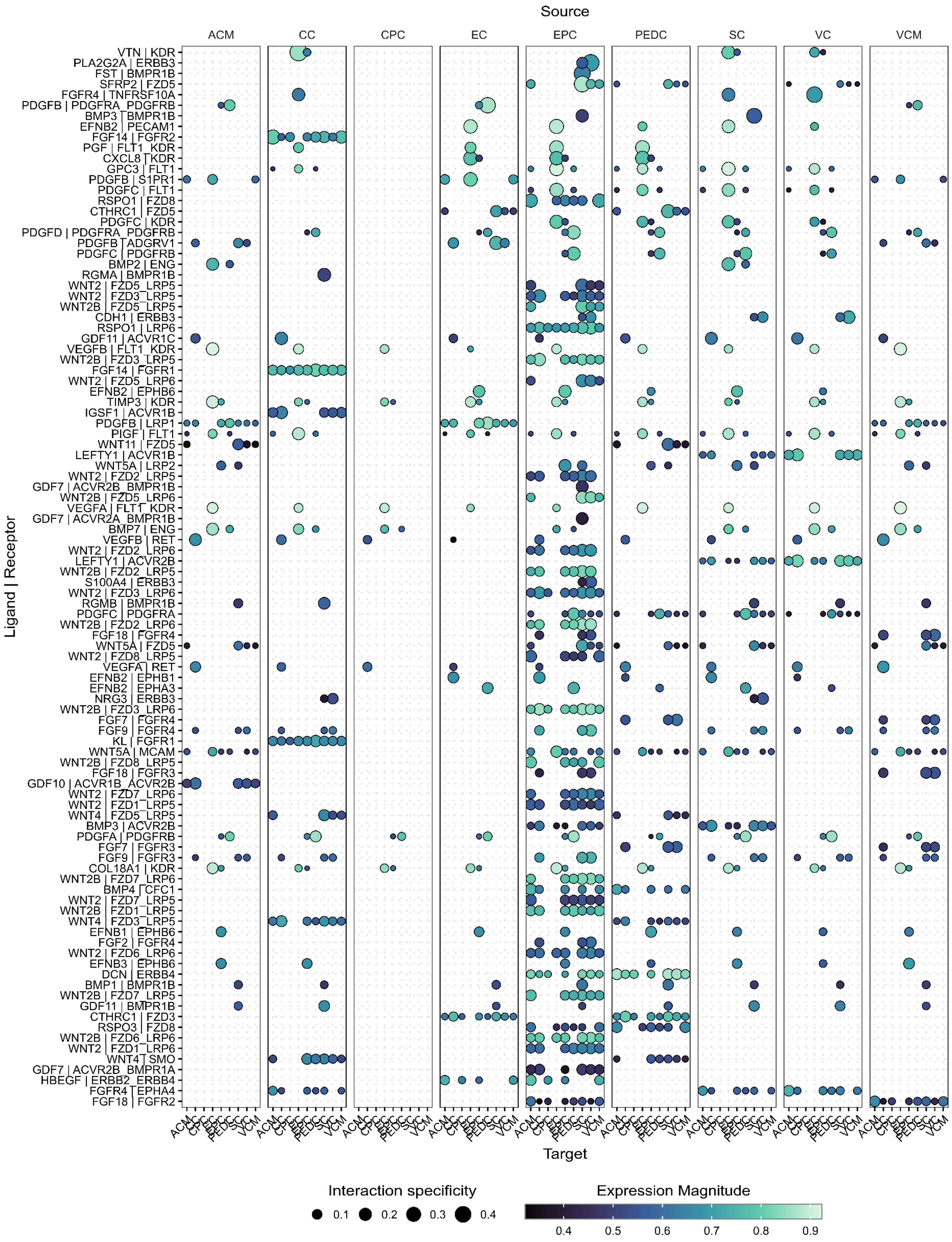

Supplementary Fig. 19. Cell-cell communication via ligand-receptor pairing for each cluster using scRNAseq data from the EMM2/1 condition.

Control

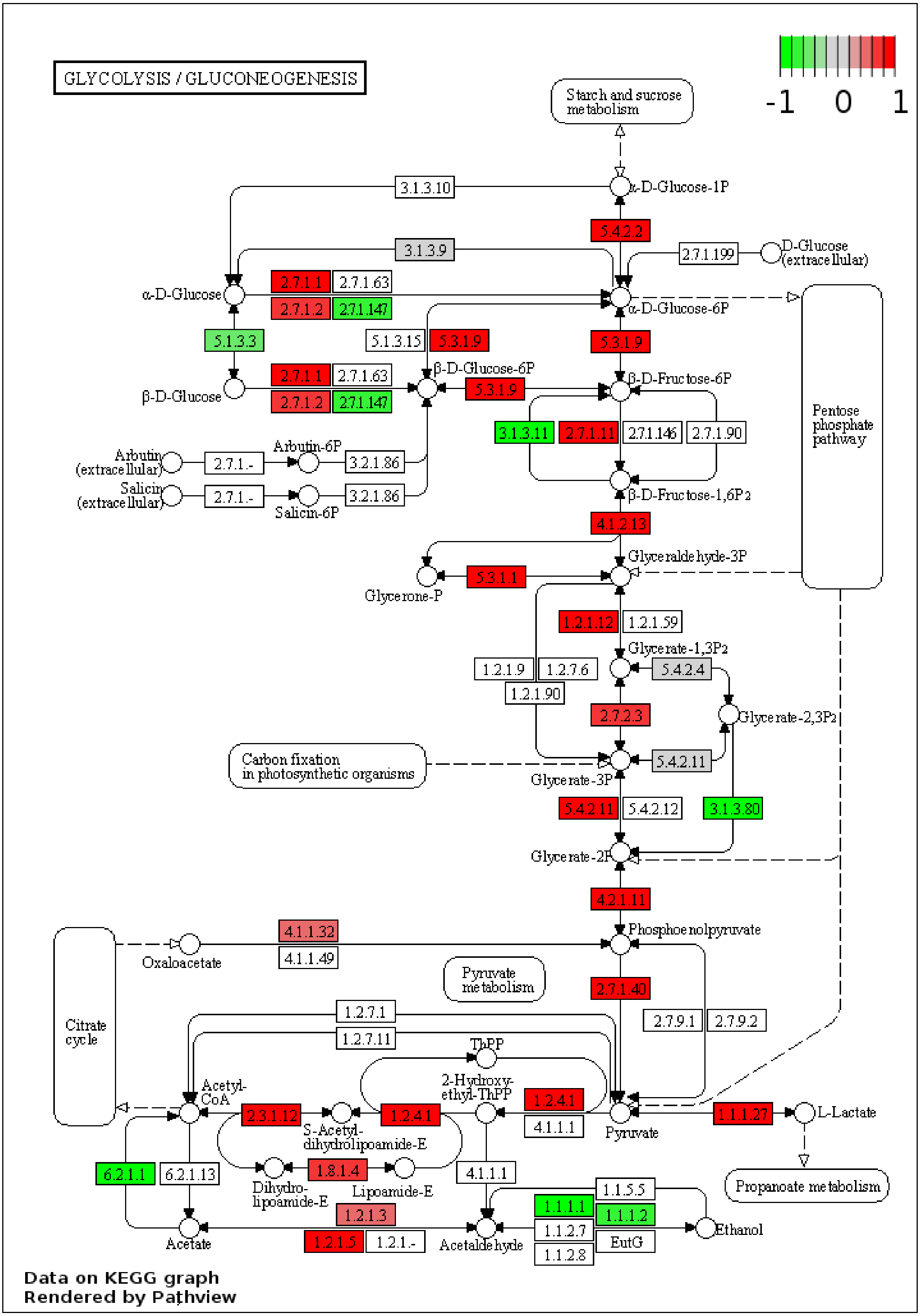

Supplementary Fig. 20. KEGG map of glycolysis and gluconeogenesis enzyme pathway rendered by Pathview. Log2-ratio calculated using scRNAseq dataset from the control condition.

MM

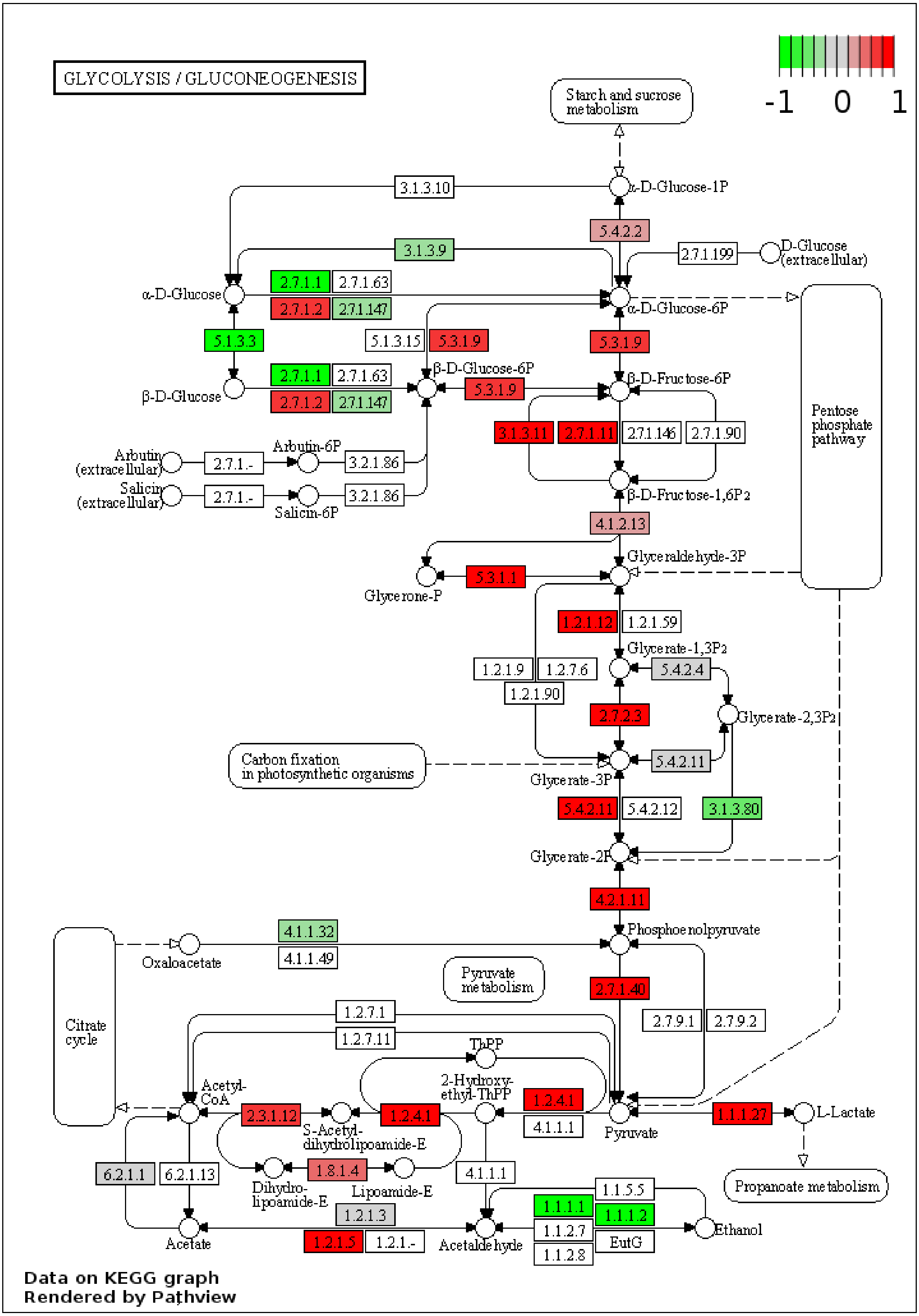

Supplementary Fig. 21. KEGG map of glycolysis and gluconeogenesis enzyme pathway rendered by Pathview. Log2-ratio calculated using scRNAseq dataset from the MM condition.

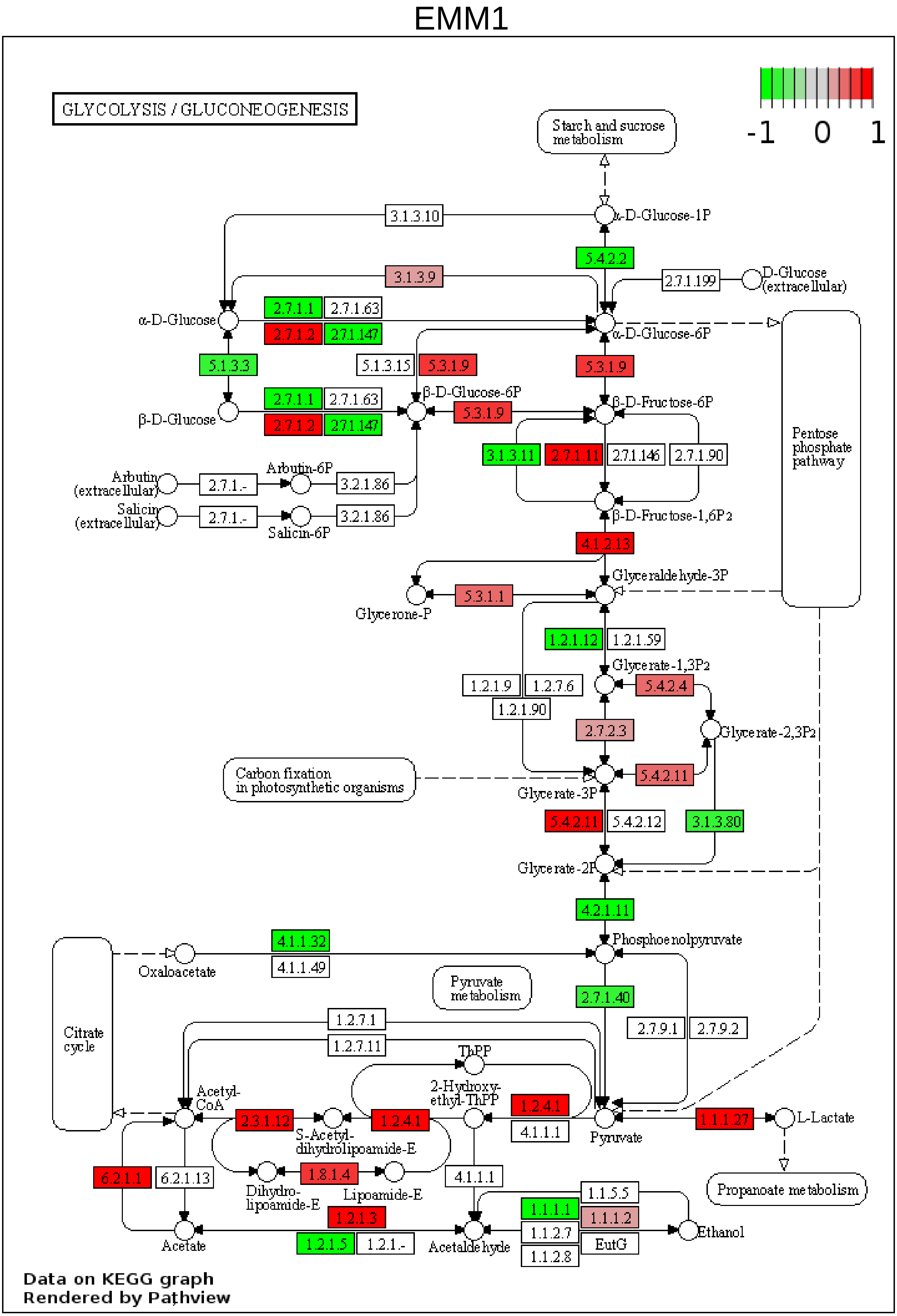

Supplementary Fig. 22. KEGG map of glycolysis and gluconeogenesis enzyme pathway rendered by Pathview. Log2-ratio calculated using scRNAseq dataset from the EMM1 condition.

EMM2/1

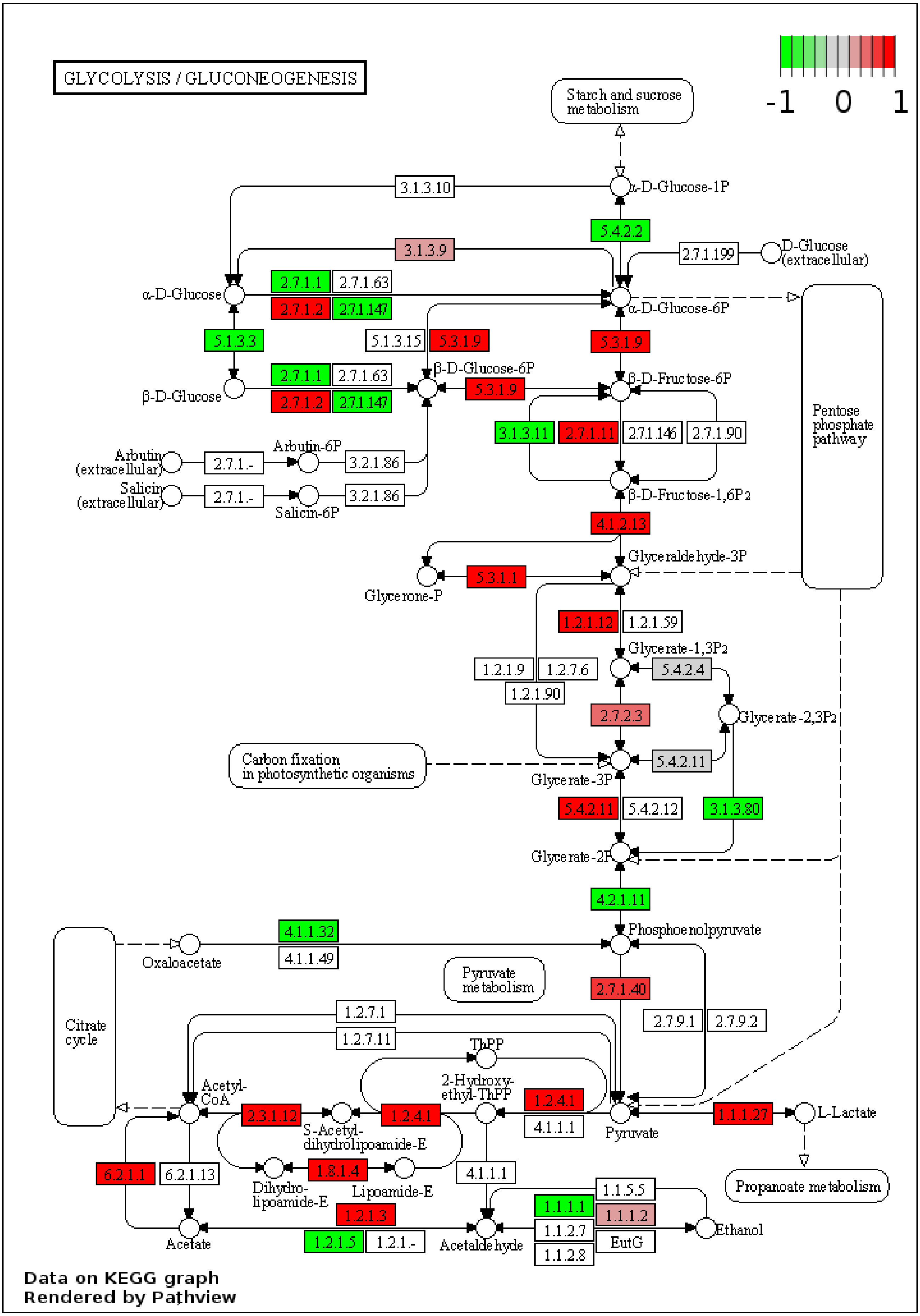

Supplementary Fig. 23. KEGG map of glycolysis and gluconeogenesis enzyme pathway rendered by Pathview. Log2-ratio calculated using scRNAseq dataset from the EMM2/1 condition.

Control

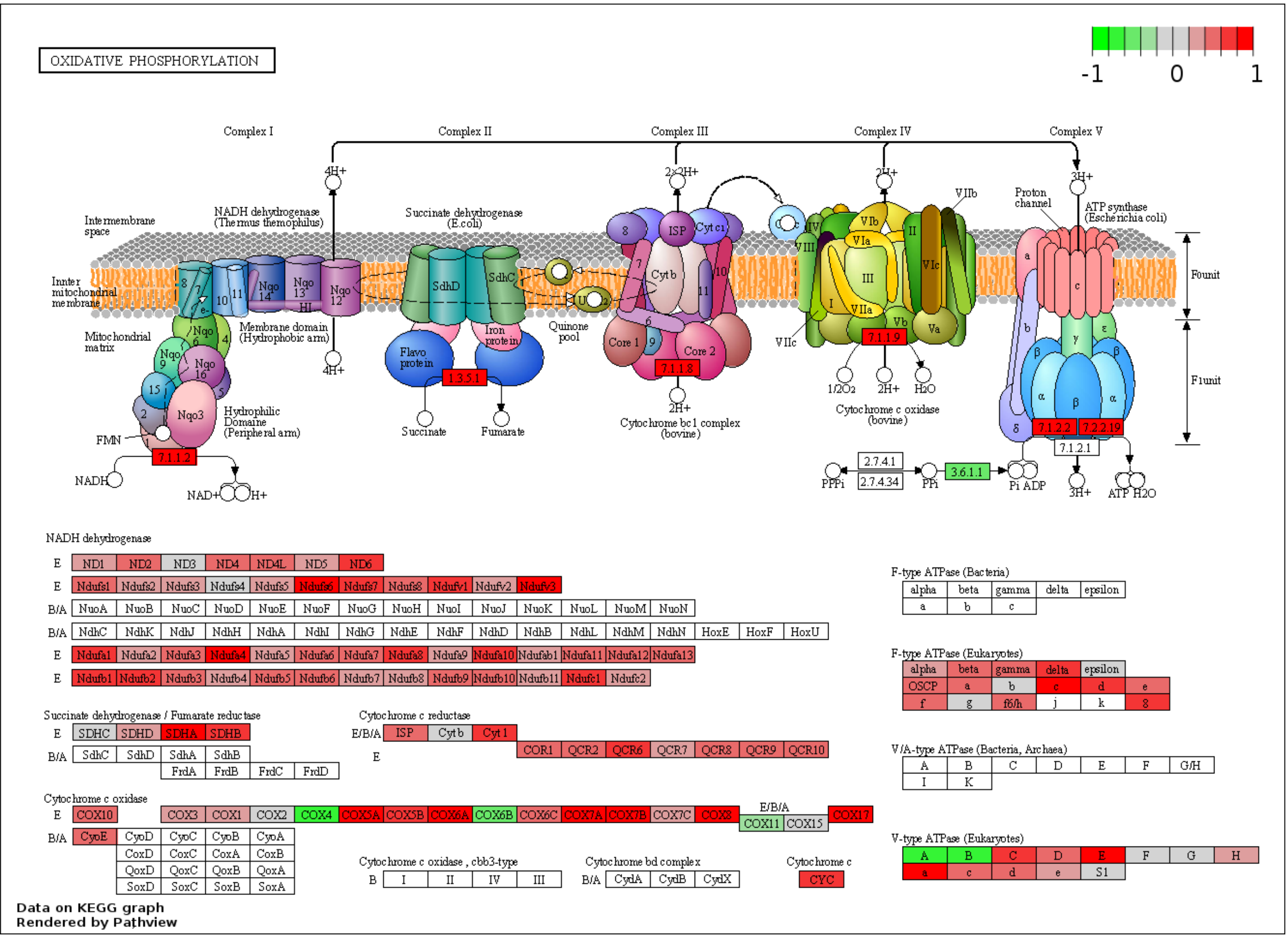

Supplementary Fig. 24. KEGG map of oxidative phosphorylation enzyme pathway rendered by Pathview. Log2-ratio calculated using scRNAseq dataset from the control condition.

MM

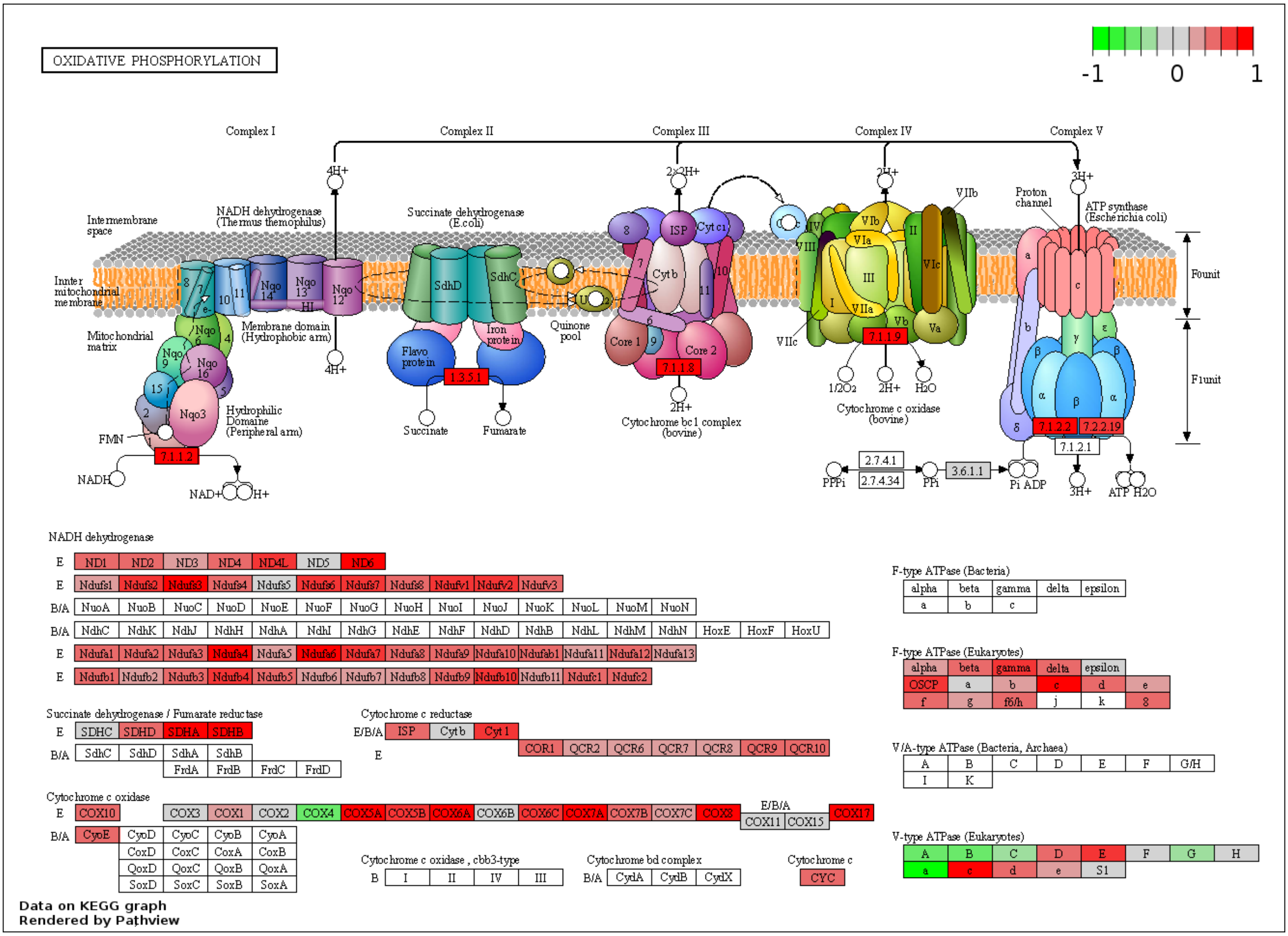

Supplementary Fig. 25. KEGG map of oxidative phosphorylation enzyme pathway rendered by Pathview. Log2-ratio calculated using scRNAseq dataset from the MM condition.

EMM1

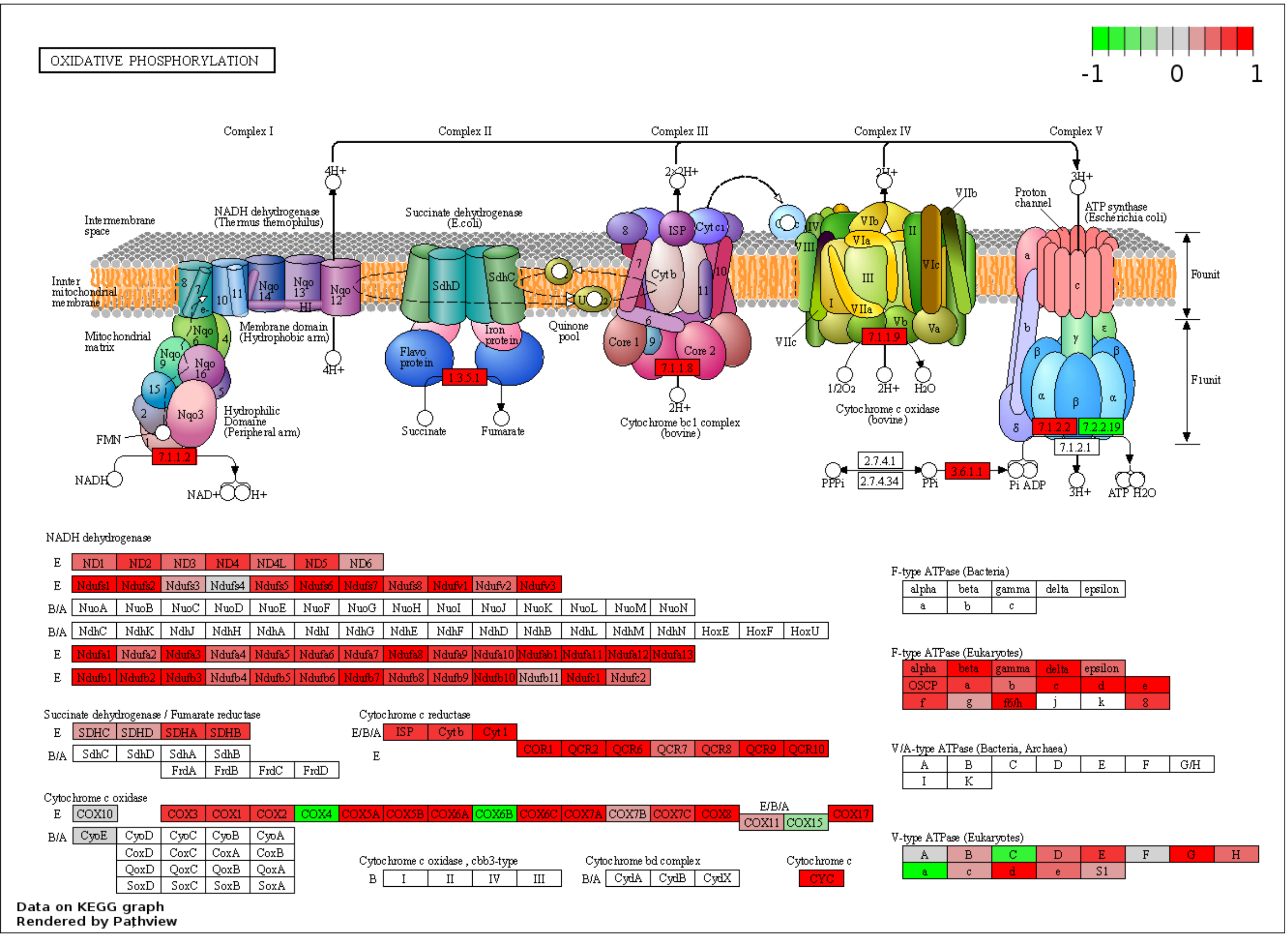

Supplementary Fig. 26. KEGG map of oxidative phosphorylation enzyme pathway rendered by Pathview. Log2-ratio calculated using scRNAseq dataset from the EMM1 condition.

EMM2/1

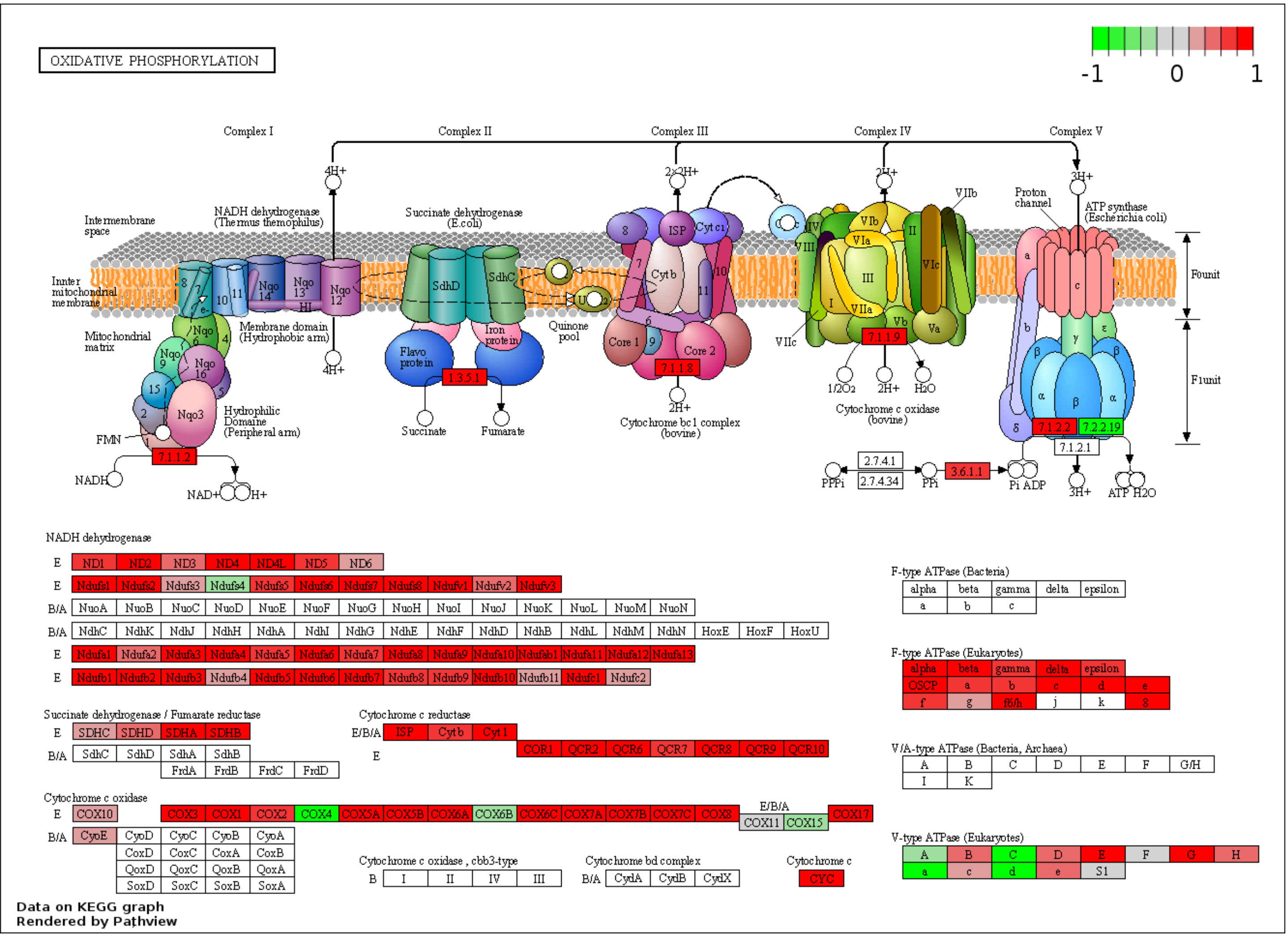

Supplementary Fig. 27. KEGG map of oxidative phosphorylation enzyme pathway rendered by Pathview. Log2-ratio calculated using scRNAseq dataset from the EMM2/1 condition.

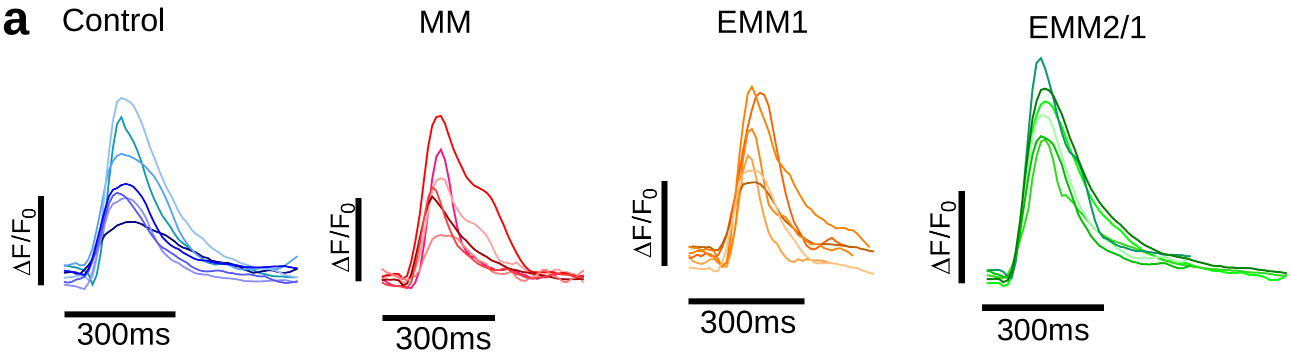

Supplementary Fig. 28. Calcium measurement displays reproducibility across independent organoids. a, Representative calcium transient traces within day 30 human heart organoids from each condition (n=7, 6, 6, and 6 independent organoids for control, MM, EMM1, and EMM2/1, respectively). Traces represent data from an individual cardiomyocyte within human heart organoids. Source data are provided as a Source Data file.

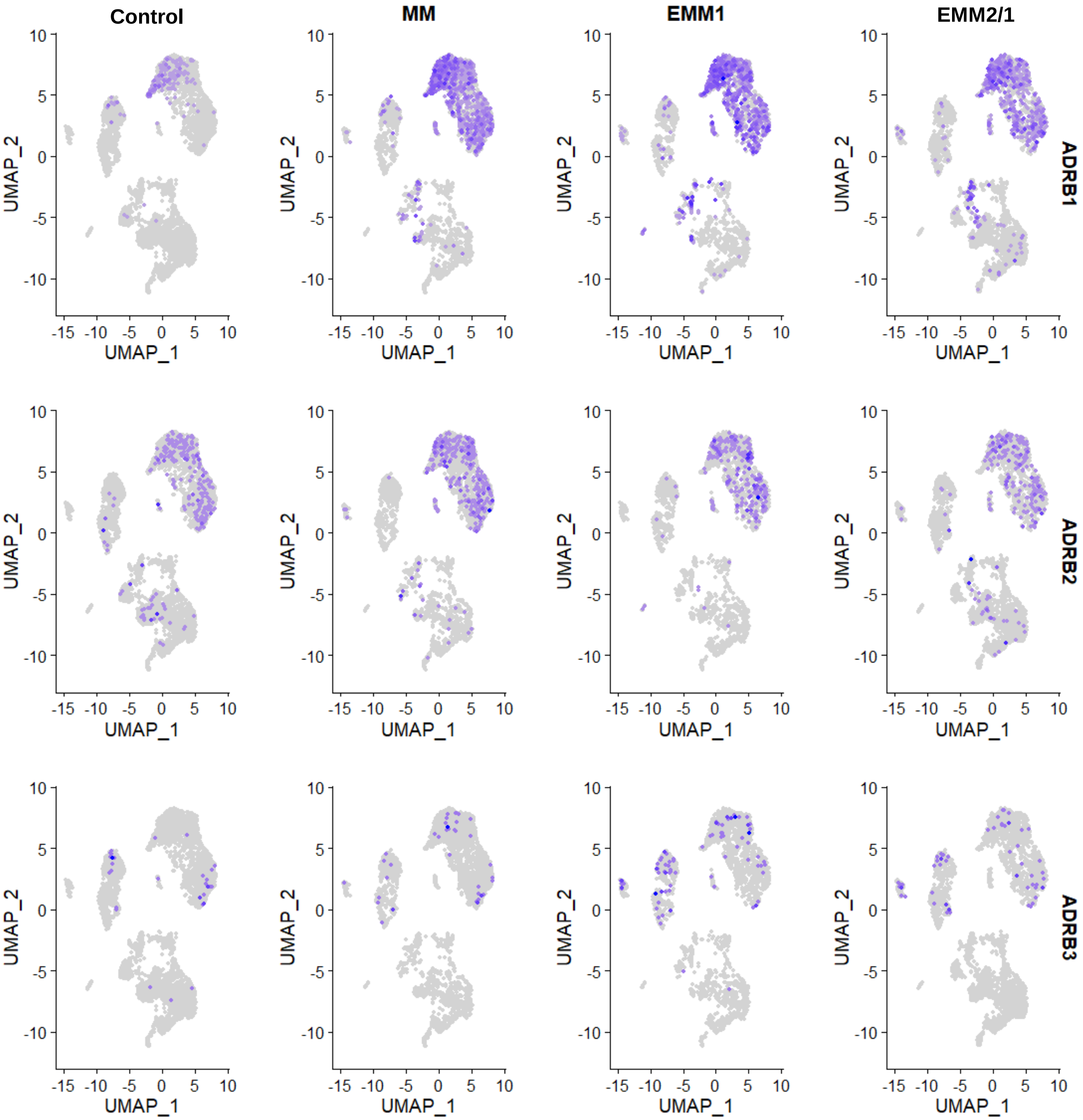

Supplementary Fig. 29. Feature plots of β-adrenoreceptor genes within single cell RNA sequencing datasets in each condition.

Supplementary Fig. 30. a, Immunofluorescence images of WGA staining (t-tubules) within TNNT2+ regions in day 30 organoids for each condition (n=8 organoids per condition). Green = WGA, TNNT2 = red, DAPI = blue). Scale bar = 20 μm. **b,** Quantification of t-tubules surrounding individual nuclei for each condition (n=8 organoids per condition). Values = mean ± s.e.m., one-way ANOVA with Dunnett's multiple comparisons tests. Source data are provided as a Source Data file.

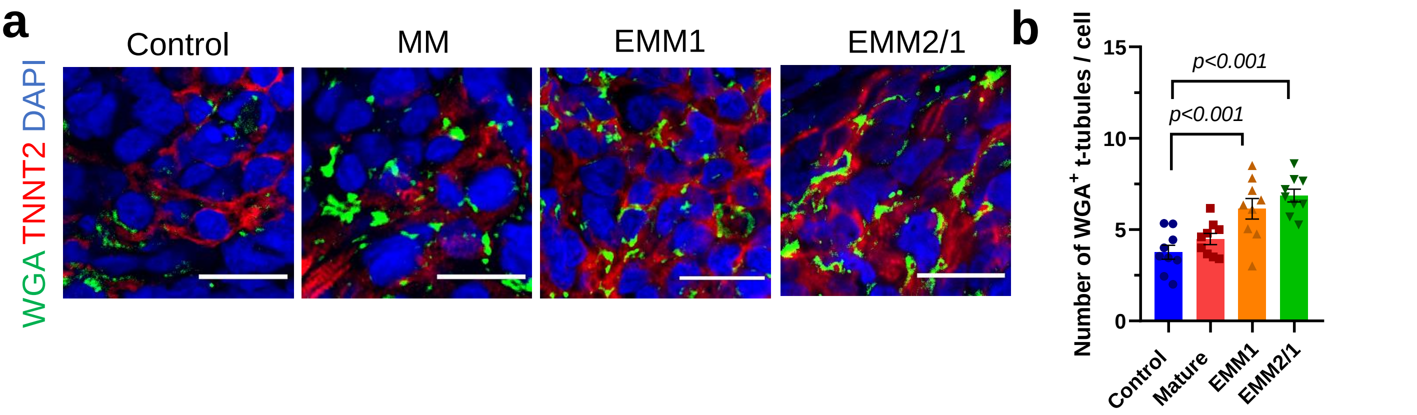

Supplementary Fig. 31. Representative immunofluorescence images of WT1+ and TNNT2+ cells at interior planes of organoids for the control and EMM2/1 conditions at Day 30 (n=12-15 independent organoids per condition across two independent experiments). WT1 = green, TNNT2 = red, DAPI = blue. Scale bar = 50 μm.

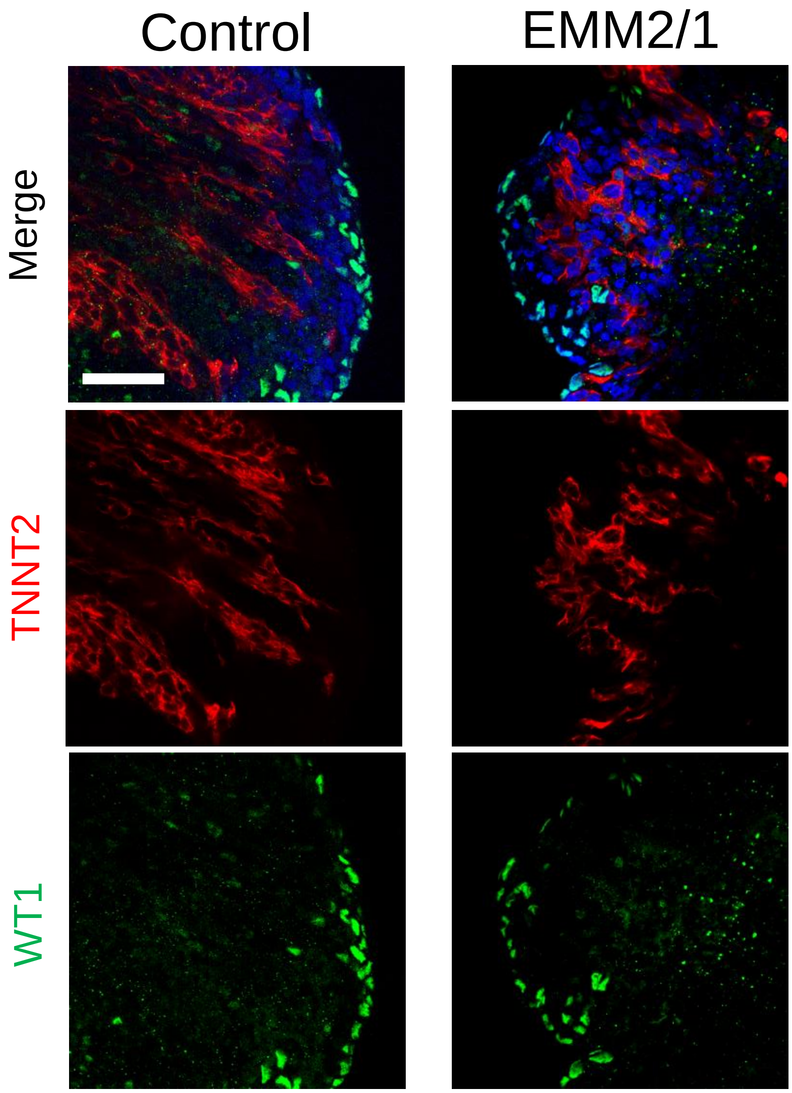

Supplementary Fig. 32. Percentage of chambered organoids at day 30 in the EMM2/1 condition across multiple cell lines (n=12 organoids for L1 organoids; n=11 organoids per condition for BYS0111 organoids; n=12 organoids per condition for H9 organoids; all across two independent experiments). Source data are provided as a Source Data file.

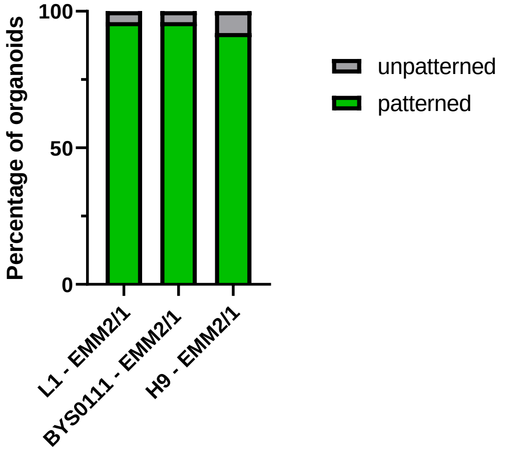

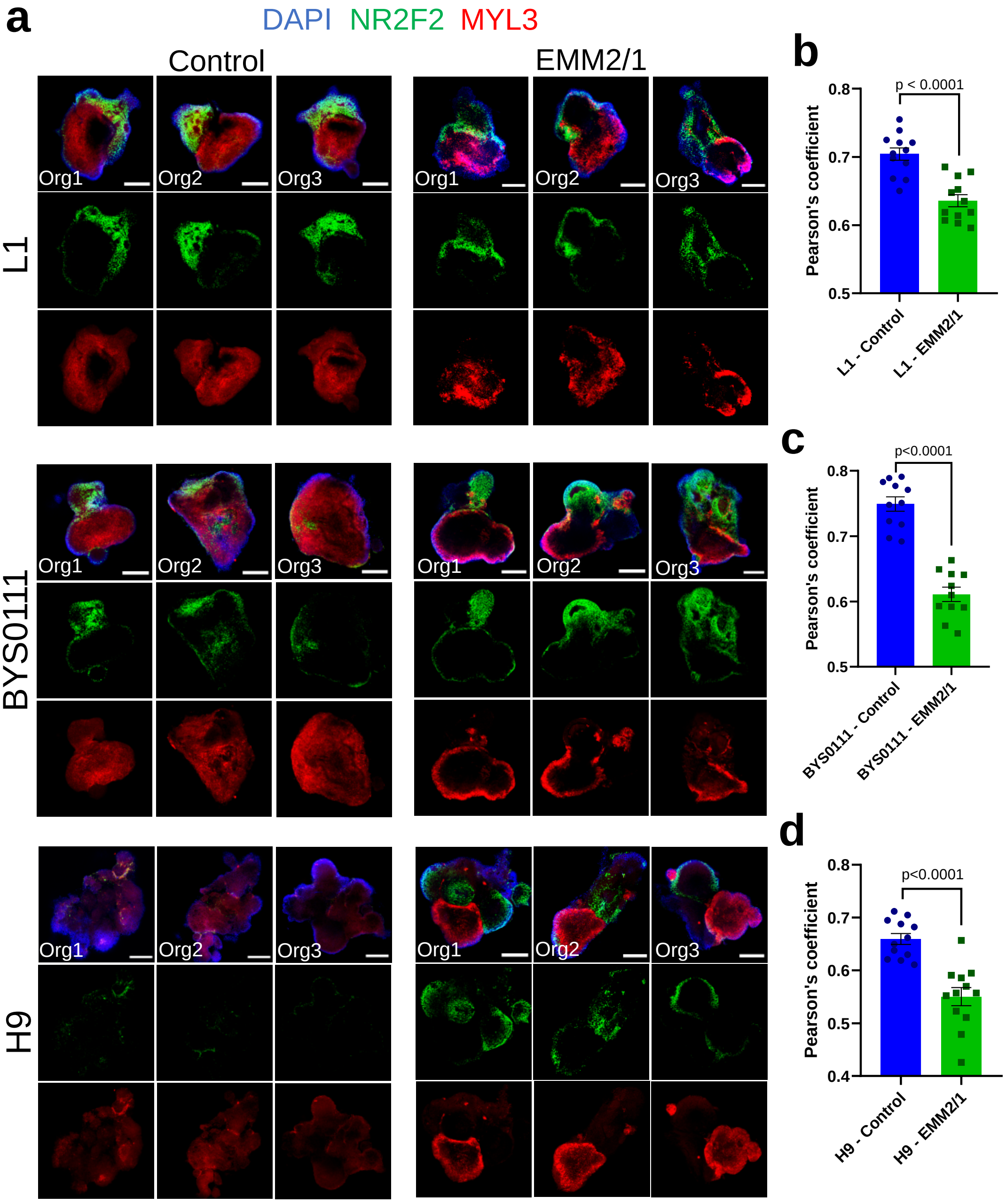

Supplementary Fig. 33. Ventricular and atrial chamber formation is reproducible across three independent hPSC lines. a, Representative immunofluorescence images of individual day 30 organoids in both Control and EMM2/1 conditions from the cell lines L1, BYS0111, and H9 displaying NR2F2 (green), MYL3 (red), and DAPI (blue). Three organoids are displayed per condition per cell line (n=12 independent organoids per condition for L1 organoids; n=11 organoids per condition for BYS0111 organoids; n=12 organoids per condition for H9 organoids; all across two independent experiments). Scale bars = 200 μm. **b-d,** Quantification of colocalization (Pearson's coefficient) between NR2F2 (green) and MYL3 (red) from images presented in **(a)** for L1 **(b)**, BYS0111 **(c)**, and H9 **(d)** organoids. Values = mean ± s.e.m., unpaired t-test. Source data are provided as a Source Data file.

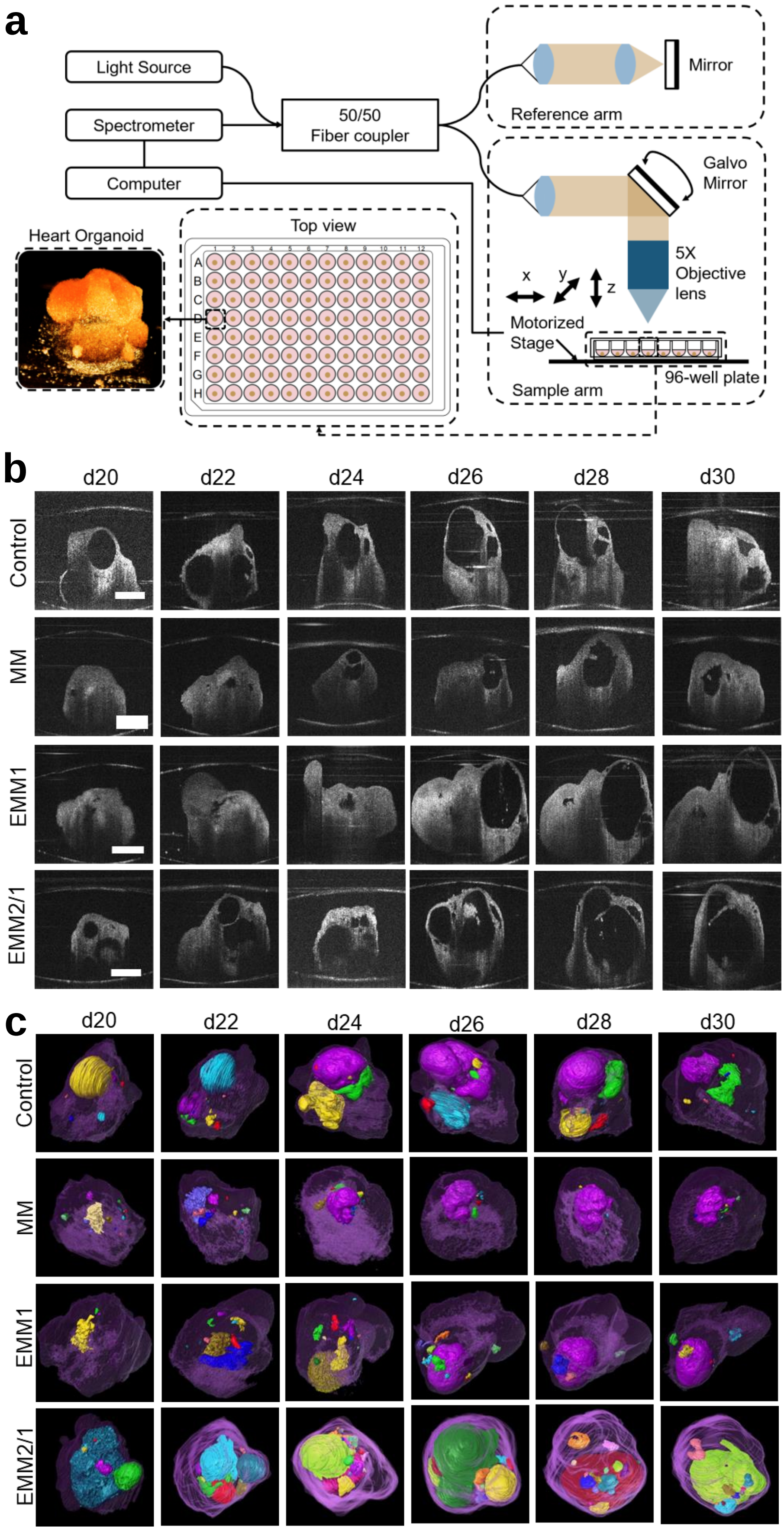

Supplementary Fig. 34. Live longitudinal imaging by optical coherence tomography reveals extent of interconnected chambers within human heart organoids. a, Schematic of custom-built optical coherence tomography (OCT) system for heart organoid imaging. Created with BioRender.com. **b,** Longitudinal OCT cross-sectional scans of heart organoids from day 20 to day 30 in each condition. Scale bars = 500 µm. Images shown represent 6 organoids per condition across two independent experiments. **c,** 3D segmentation of OCT scans from images presented in **(a)** reveal the temporally dynamic volumetric visualization of chamber identity in each condition.

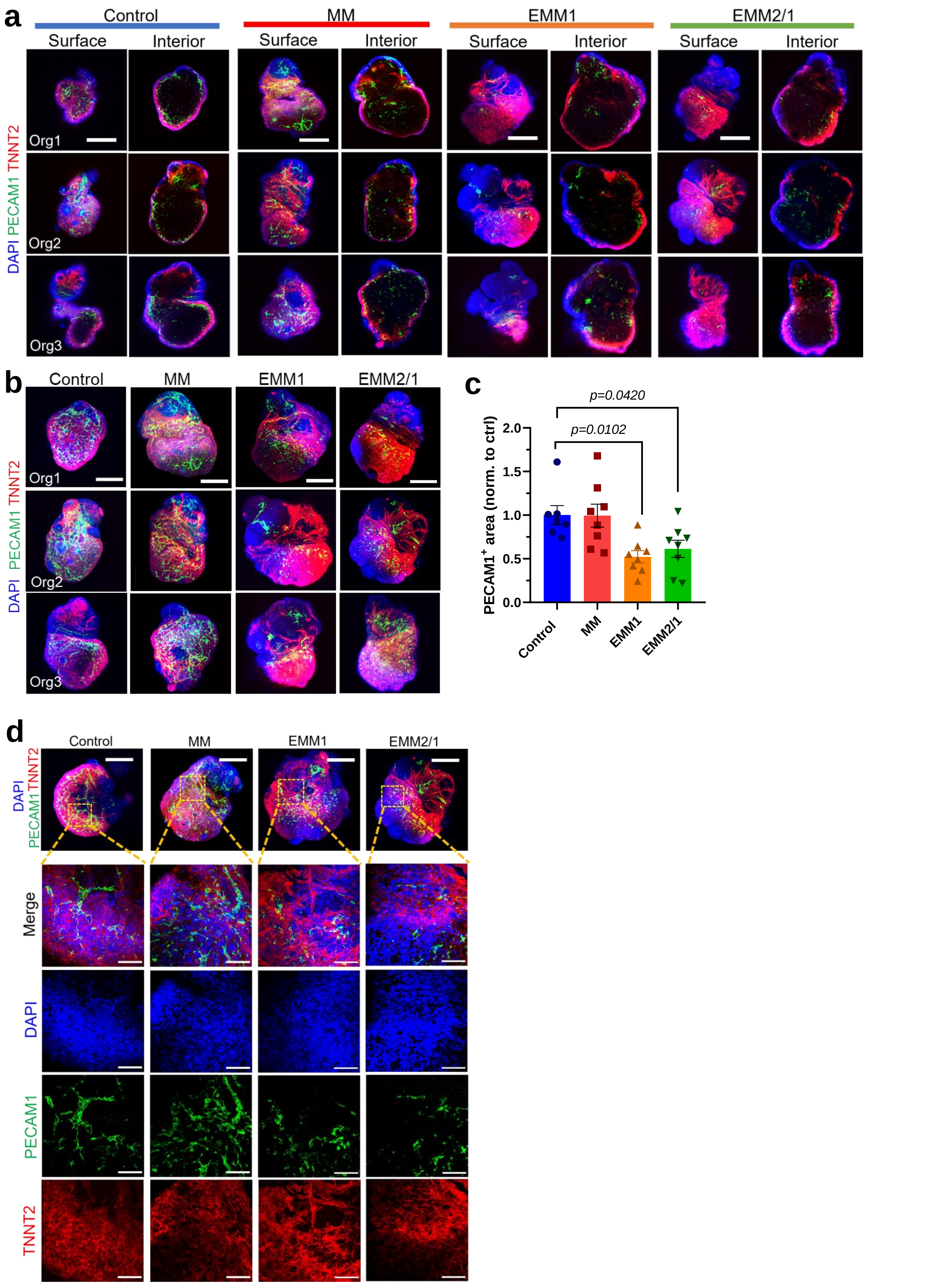

Supplementary Fig. 35

Supplementary Fig. 35. Endothelial cell localization and morphology is perturbed through enhanced developmental maturation strategies. a, Representative immunofluorescence images from the surface and interior of day 30 organoids with DAPI (blue), TNNT2 (red), and PECAM1 (green) in each condition (n=7-8 independent organoids per condition across two independent experiments).Scale bars = 200 µm. **b,** Representative day 30 organoid immunofluorescence images with DAPI (blue), TNNT2 (red), and PECAM1 (green) from images presented in **(a)** (n=7, 8, 8, and 8 independent organoids for control, MM, EMM1, and EMM2/1, respectively). Images presented as maximum intensity projections. Scale bars = 200 µm. **c,** Quantification of PECAM1+ area presented in **(b)** (n=7, 8, 8, and 8 independent organoids for control, MM, EMM1, and EMM2/1, respectively). Data presented as log fold change normalized to control. Values = mean ± s.e.m., one-way ANOVA with Dunnett's multiple comparisons test. **d,** Representative high magnification immunofluorescence images of organoids in each condition with DAPI (blue), TNNT2 (red), and PECAM1 (green(n=7, 8, 8, and 8 independent organoids for control, MM, EMM1, and EMM2/1, respectively). Scale bars = 50 µm. The images on top are representative low magnification organoids (Scale bar = 200 µm) for each condition with the yellow square representing area of high magnification. Images presented as maximum intensity projections. Source data are provided as a Source Data file.

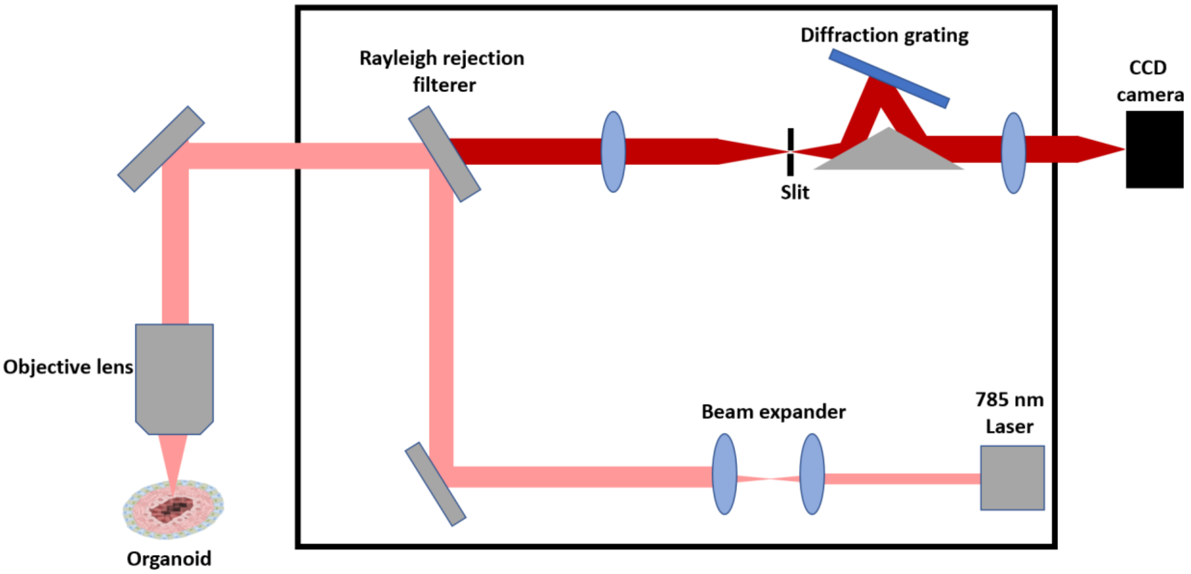

Supplementary Fig. 36. Schematic diagram for the Renishaw confocal Raman spectrometer for use with human heart organoids. Created with BioRender.com.

Supplementary Fig. 37. Representative immunofluorescence images of individual day 30 organoids in the MM and EMM1 conditions displaying ALDH1A2 (green), epicardial marker TBX18 (red), and DAPI (blue). Three organoids are displayed for each condition (representative of n=22-24 independent organoids per condition across three independent experiments). Scale bar = 200 μm.

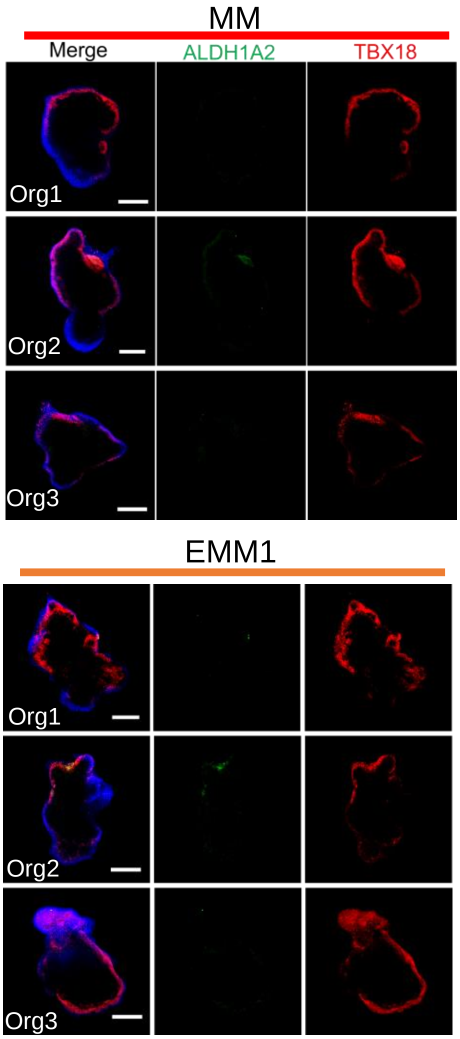

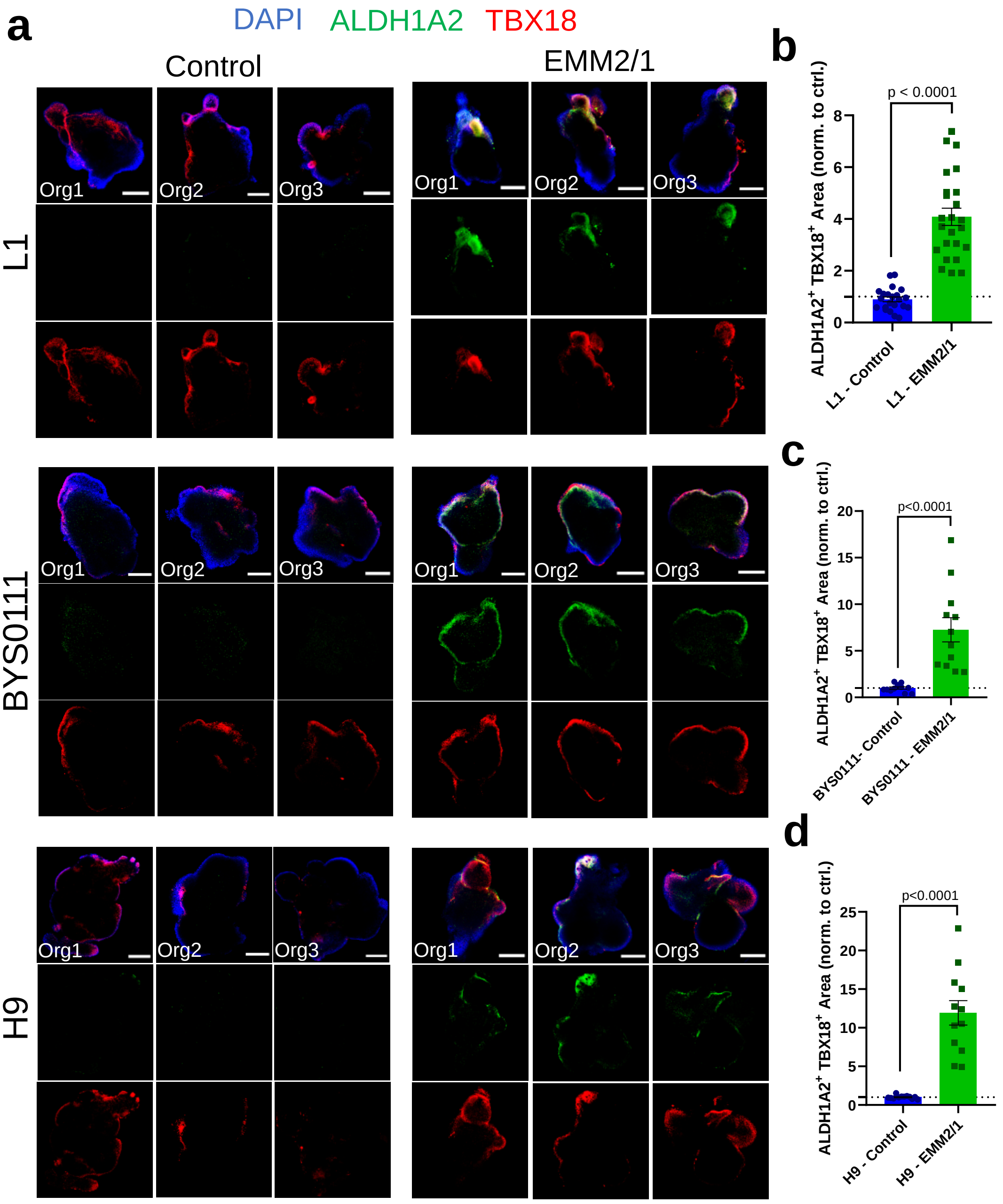

Supplementary Fig. 38. Emergence of an ALDH1A2+ proepicardial pole through using the EMM2/1 developmental maturation strategy is reproducible across three hPSC lines. a, Representative immunofluorescence images of individual day 30 organoids in both Control and EMM2/1 conditions from the cell lines L1, BYS0111, and H9 displaying ALDH1A2 (green), TBX18 (red), and DAPI (blue). Three organoids are displayed per condition per cell line (n=22 or 24 independent organoids for control or EMM2/1, respectively, for L1 organoids across three independent experiments; n=12 organoids per condition for BYS0111 organoids across two independent experiments; n=12 organoids per condition for H9 organoids across two independent experiments). Scale bars = 200 μm. **b-d,** Quantification of ALDH1A2^{+} TBX18^{+} area within organoids in each condition from images presented in **(a)** for L1 **(b)**, BYS0111 **(c)**, and H9 **(d)** organoids. Data presented as fold change normalized to Control. Values = mean ± s.e.m., unpaired t-test. Source data are provided as a Source Data file.

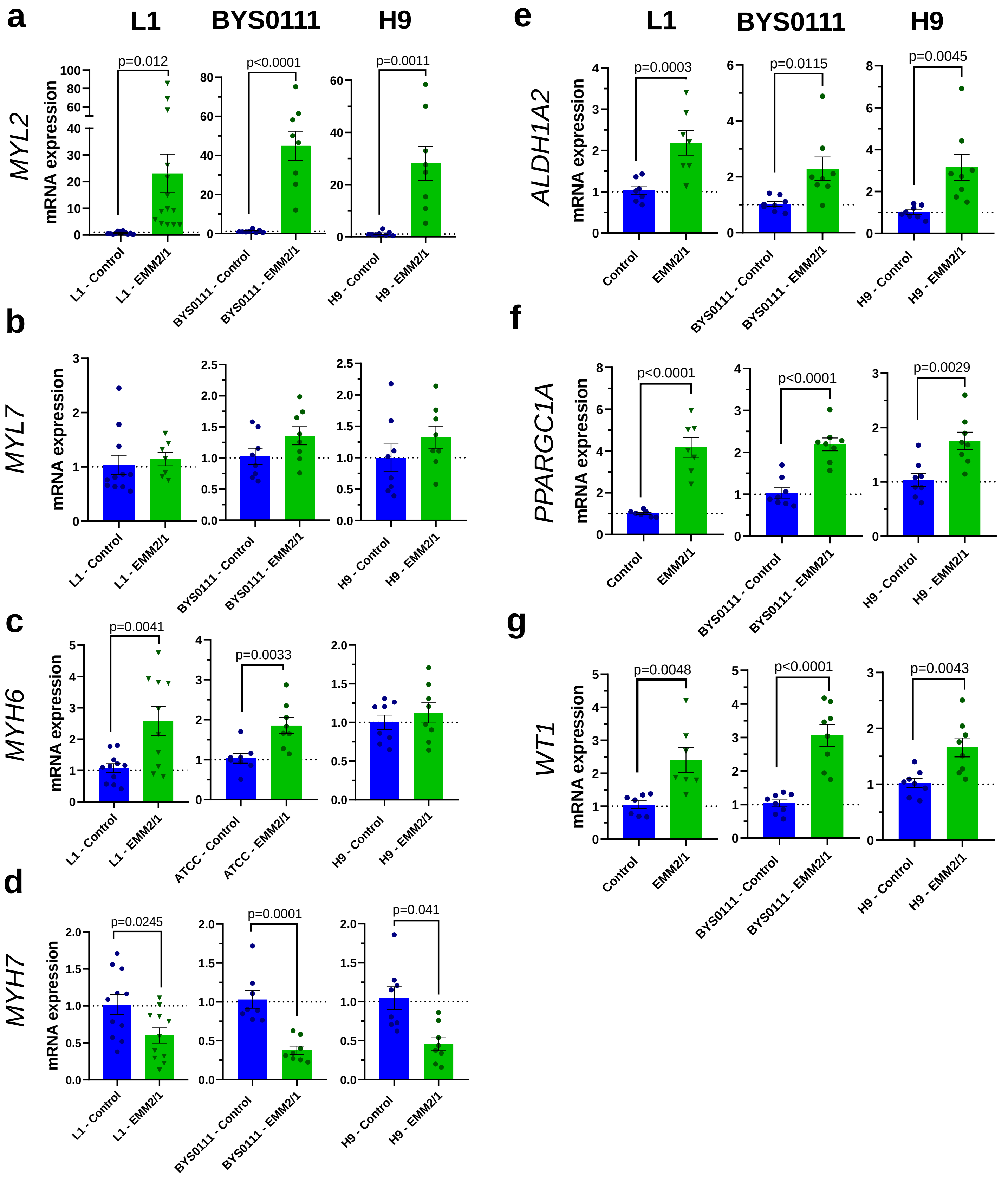

Supplementary Fig. 39. Transcriptional profiles for key genes within human heart organoids are reproducible across three hPSC lines. (a-g,) mRNA expression of select genes in the Control and EMM2/1 conditions from L1, BYS0111 and H9 organoids at day 30 (n=7-14 independent organoids per condition for L1 organoids across three independent experiments; n=8 organoids per condition for BYS0111 organoids across two independent experiments; n=8 organoids per condition for H9 organoids across two independent experiments). Genes presented are *MYL2* **(a)**, *MYL7* **(b)**, *MYH6* **(c)**, *MYH7* **(d)**, *ALDH1A2* **(e)**, *PPARGC1A* **(f)**, and *WT1* **(g)**. Data presented as $\log_2$ fold change normalized to Control for each cell line. Values = mean ± s.e.m., unpaired t-test. Source data are provided as a Source Data file.

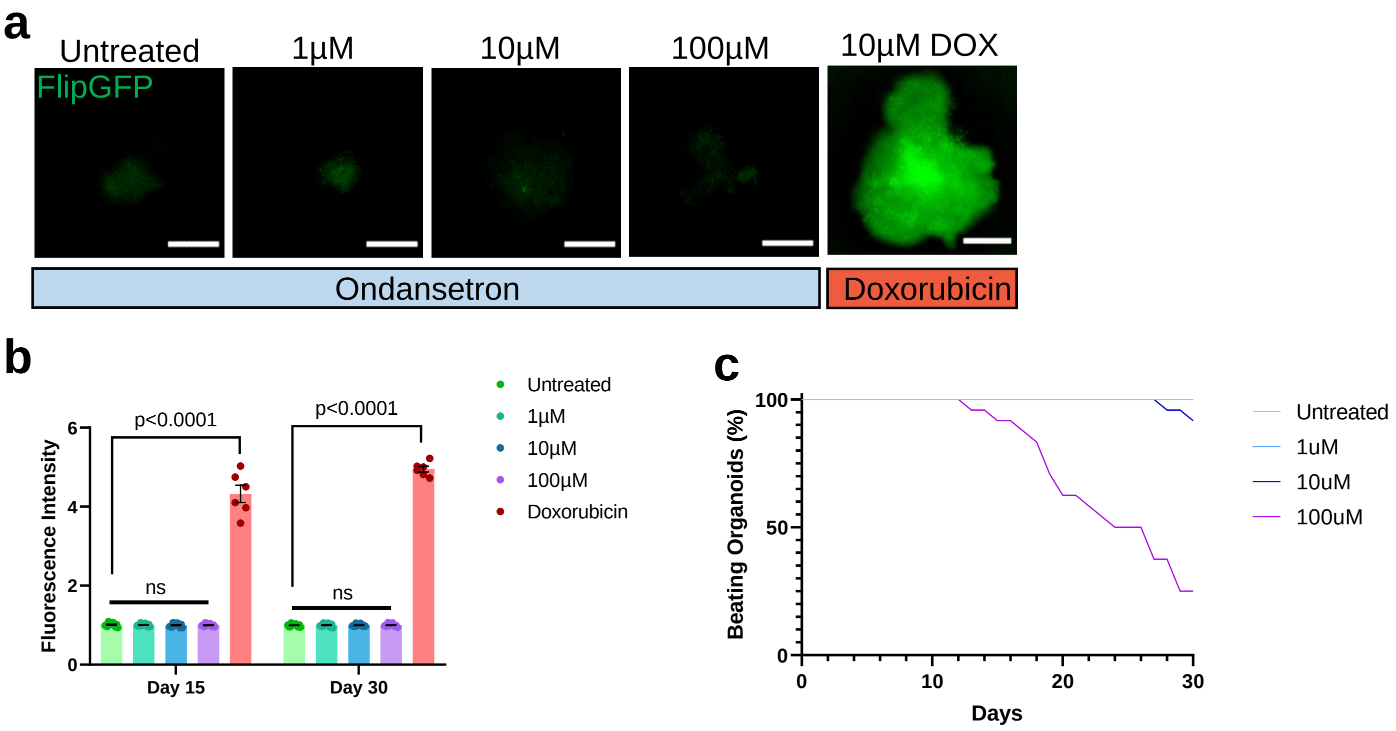

Supplementary Fig. 40. Apoptosis is not a contributing factor towards ondansetron-induced cardiac defects. a, Representative fluorescence images of day 30 EMM2/1 organoids from each condition displaying FlipGFP fluorescence signal following Ondansetron treatment from day 9 to day 30 and Doxorubicin treatment from day 28 to day 30 (across two independent experiments each: n=12 independent organoids per condition; n=6 independent organoids for doxorubicin group). Scale bars = 200 μm. **b,** Quantification of fluorescence intensity from images presented in **(a)** and from organoids at day 15 (across two independent experiments each: n=12 independent organoids per condition; n=6 independent organoids for doxorubicin group). Data presented as fold change normalized to Untreated (Day 30). Values = mean ± s.e.m., matched two-way ANOVA with multiple comparisons. **c,** Quantification of percentage of beating organoids throughout treatment period for each Ondansetron condition from day 0 to day 30 (n=24 independent organoids per condition across two independent experiments). Source data are provided as a Source Data file.

Supplementary Fig. 41. Schematic for **(a)** the measurement of organoid chamber area and **(b)** area of positive fluorescence signal. Plane for measurement was selected to be the middle plane of the organoid (50% of organoid thickness as measured by confocal microscopy).

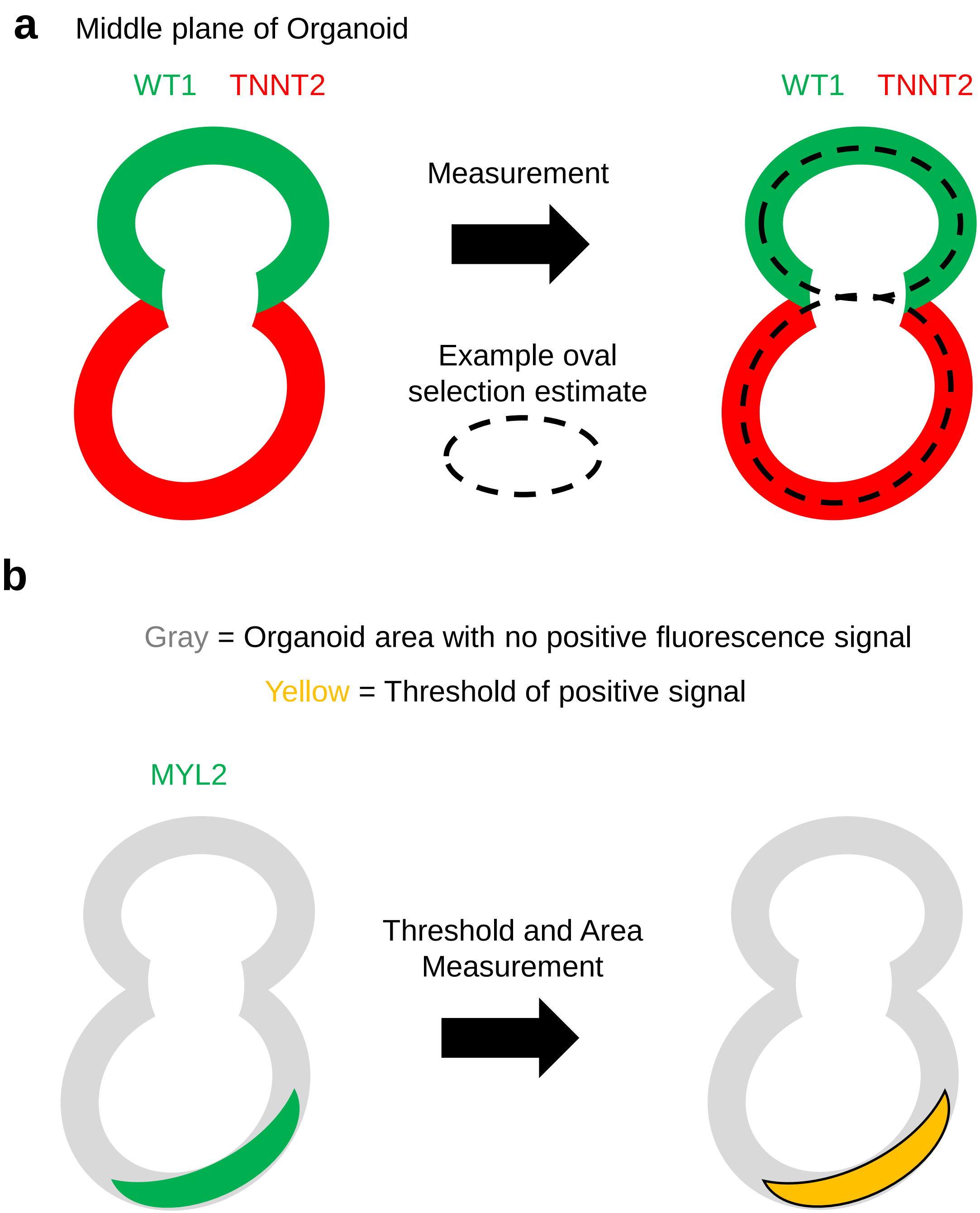

Supplementary Table 1. Antibodies used for immunofluorescence.

	Antibody Name	Host Species	Dilution	Catalogue Number	Vendor
Primary	TNNT2	Mouse	1:200	ab8295	Abcam
	TNNT2	Rabbit	1:200	ab45932	Abcam
	WT1	Rabbit	1:200	ab89901	Abcam
	PECAM1	Mouse	1:50	P2B1	DSHB
	MYL2	Rabbit	1:200	ab79935	Abcam
	MYL7	Mouse	1:200	311-011	Synaptic Systems
	TBX18	Mouse	1:200	ab201587	Abcam
	ALDH1A2	Rabbit	1:200	ABN420	Sigma
	KCNJ2	Rabbit	1:500	HPA029109	Sigma
	NR2F2	Rabbit	1:100	ab211777	Abcam
	MYL3	Mouse	1:50	Sc-47719	Santa Cruz Biotechnology
	Caveolin-3	Mouse	1:50	MAB6706-SP	R&D Systems
Secondary	Alexa Fluor 488	Donkey anti-mouse	1:200	A-21202	Thermo Fisher Scientific
	Alexa Fluor 488	Donkey anti-rabbit	1:200	A-21206	Thermo Fisher Scientific
	Alexa Fluor 594	Donkey anti-mouse	1:200	A-21203	Thermo Fisher Scientific
	Alexa Fluor 594	Donkey anti-rabbit	1:200	A-21207	Thermo Fisher Scientific
	Alexa Fluor 647	Donkey anti-rabbit	1:200	A-31573	Thermo Fisher Scientific

SUPPLEMENTARY METHODS

*Heart organoid dissociation***.** Organoids were collected on day 30 from each maturation strategy (control, MM, EMM1, EMM2/1). Organoids were individually placed into separate 1.5mL microcentrifuge tubes (Eppendorf), dissociated and pooled. Organoids were dissociated into a single-celled suspension using a modified protocol of the STEMdiff Cardiomyocyte Dissociation Kit (STEMCELL Technologies). Upon being transferred to a microcentrifuge tube, organoids were washed with PBS, submerged in 200 µL of warm dissociation media (37 ° C), and placed on a thermal mixer at 37 ° C and 300rpm for 5 minutes. Then, the supernatant was collected and transferred to a 15 mL falcon tube (Corning) containing 5mL of respective media (control, MM, EMM1, etc.) containing 2% BSA (Thermo Fisher Scientific). An additional 200 µL of warm dissociation media (37 ° C) was then added back to the organoid on a thermal mixer (37 ° C). The organoid dissociation media solution was then pipetted up and down gently 3-5 times. The organoid was allowed to sit on the thermal mixer for an additional 5 minutes. If the organoid remained visible, the process was repeated. Once the organoid was no longer visible, the microcentrifuge tube solution was pipetted up and down gently 3-5 times and its entire contents were transferred to the 15 mL falcon tube containing the respective media + 2% BSA and cells. These tubes were then centrifuged at 300 *g* for 5 minutes. The supernatant was aspirated, and the cell pellets were resuspended in respective media + 2% BSA. Using a hemocytometer, viability, cell counts, and aggregate percentage were acquired. 4 organoids were pooled per condition for scRNA-seq. Cell viability was then assessed using trypan blue (Gibco) mixed with the cell solution at a 1:1 volume ratio and was incubated for 5 minutes at room temperature. A hemocytometer was then used to count the cells and to assess viability.

Transmission Electron Microscopy (TEM). Human heart organoids were fixed on Day 15 and Day 30 for each condition in 2.5% glutaraldehyde (Electron Microscopy Solutions) in PBS for 45 minutes, washed three times in PBS for 5 minutes each, then stored at 4 ºC. Samples were then washed with 100mM phosphate buffer and postfixed with 1% osmium tetroxide in 100mM phosphate buffer, dehydrated in a gradient series of acetone and infiltrated and embedded in Spurr (Electron Microscopy Sciences). 70 nm thin sections were obtained with a Power Tome Ultramicrotome (RMC, Boeckeler Instruments. Tucson, AZ) and post stained with uranyl acetate and lead citrate. A JEOL 1400Flash Transmission Electron Microscope (Japan Electron Optics Laboratory, Japan) was used to acquire images at an accelerating voltage of 100k. Data was processed using Fiji. 16 total slices from 4 organoids per condition were used in the assessment and quantification of sarcomere length and mitochondrial size. Sarcomere length was measured using the straight-line tool between Z disks. Mitochondrial area was measured using the freehand selection tool.

Calcium imaging. Calcium transient activity within the human heart organoids was assessed using Fluo-4 AM (Thermo Fisher Scientific). Fluo-4 AM was solubilized in DMSO per the manufacturer's instructions. A 1.5 μM solution of Fluo-4 was prepared in the corresponding medium (control, MM, EMM1, EMM2/1). Organoids were washed twice using RPMI 1640 basal medium, then Fluo-4 AM was added at a final concentration of 1 μM and incubated for 30 minutes at 37 °C and 5% CO_2. Organoids were then washed twice using their respective medium (control, MM, EMM1, etc.) and transferred to a chambered coverglass slide (Cellvis) using a cut 200 μL pipette tip. Videos were acquired using a Cellvivo microscope (Olympus) at 100X magnification at 100 frames per second over 10 total seconds as an image stack. Samples were excited at 494 nm excitation and 506 nm emission was collected. Data was processed using Fiji and Microsoft Excel. Individual cardiomyocytes from the acquired images were randomly selected using the freehand selection tool, and the mean gray value over time was quantified. When quantifying amplitude and frequency, a minimum of 2 regions and 16 peaks were quantified and averaged for each organoid. Baseline F_0 of fluorescence intensity F was calculated using the average of the lowest 50 intensity values in the acquired dataset. Fluorescence change $\Delta F/F_0$ was calculated using the equation:

- $$\frac{\Delta F}{F_0} = \frac{(F - F_0)}{F_0} \quad (1)$$

Voltage imaging. Voltage activity within human heart organoids was assessed using di-8-ANEPPS (Thermo Fisher Scientific). Di-8-ANEPPS was solubilized in DMSO per the manufacturer's instructions. A 15 μM solution of di-8-ANEPPS was prepared in respective medium (control, MM, EMM1, etc.). Organoids were washed twice using RPMI 1640 basal medium, then di-8-ANEPPS was added at a final concentration of 10 μM and incubated for 30 minutes at 37 °C and 5% CO_2. Organoids were then washed twice using their respective medium (control, MM, EMM1, etc.) and transferred to a chambered coverglass slide (Cellvis) using a cut 200 μL pipette tip. Videos were acquired using a Cellvivo microscope (Olympus) at 100 frames per second over 10 total seconds as an image stack at either 20X magnification (ondansetron) or 100X magnification (maturation electrophysiology). Samples were excited at 465 nm excitation and 630 nm emission was collected. Data was processed using Fiji and Microsoft Excel. At 20X, the whole organoid was captured, and at 100X, areas with dozens of cardiomyocytes were captured, which were then randomly selected and quantified in the analysis pipeline. Baseline F_0 of fluorescence intensity F was calculated using the average of the lowest 50 intensity values in the acquired dataset. Fluorescence change $\Delta F/F_0$ was calculated using the same method as with Calcium imaging presented above. The area of interest was selected using the freehand selection tool, then the mean gray value over time was quantified. APD30 and APD90 were measured from the midpoint of the upstroke until 30% or 90% repolarization, respectively.

Lentiviral Transduction. HEK293T (Horizon Inspired Cell Solutions) cells were transfected with the Flip-GFP plasmid (VectorBuilder) and the packaging plasmids pMD2 and psPAX2 using lipofectamine with Plus reagent (Thermo) to create a lentivirus. The lentivirus was added to iPSC-L1 cells with 8 μg/ml polybrene (Fisher Scientific) and incubated overnight. Puromycin selection was carried out for 3–5 days until all cells lacking lentivirus were absent from the well. Surviving clones were selected, collected, replated, and further expanded to give rise to the FlipGFP line.

Optical coherence tomography. A Spectral-Domain Optical Coherence Tomography (SD-OCT) system was used for label-free longitudinal imaging of the heart organoids. A superluminescent diode (EXALOS, EXC250023-00) was used as the light source with a center wavelength of ~1300 nm and a 3 dB spectrum range of ~180 nm. A spectrometer (Wasatch Photonics, Cobra 1300) based on a 2048-pixel InGaAs line-scan camera (Sensors Unlimited, GL2048) was used to provide a maximum A-scan rate of 147 kHz. A 5X objective lens was used and the transverse and axial resolutions were measured to be ~2.8 μm and ~3.0 μm in tissue, respectively. Longitudinal 3D OCT imaging was performed every other day from Day 20 to Day 30 on iPSC-L1 organoids. Each 3D OCT scan comprised 600 A-scans per B scan and 600 B-scans. Each organoid required ~22 seconds for image acquisition using an exposure time of ~40 μs for each A-scan. Eight organoids from each group were imaged and used for analysis. The media level in each well was adjusted during imaging to reduce image artifacts and minimize light absorption. Re-scaling of acquired OCT images was performed using ImageJ (NIH) to obtain isotropic pixel size in all three dimensions. Registration of the same organoids on different days, cavity segmentation, and 3D rendering were performed using Amira software (Thermo Fisher Scientific). The total volume and cavities inside the organoids were quantified from the segmentation data.

Seahorse Metabolic Assay. An Agilent Seahorse XFe96 (Agilent) was used to perform real-time extracellular flux assays. The day before the assay, 200μL of XF Calibrant was loaded into each well of the 96-well utility plate included with the sensor cartridge and the sensors were submerged in a 37 ^oC non-CO_2 incubator overnight. The day before the assay, poly-lysine (Sigma) was used to coat XFe96 spheroid microplates. In brief, poly-lysine was prepared at 100 μg/mL in water and 30 μL of this solution was added to each well of the microplate. After sitting for 20 minutes, the poly-lysine solution was aspirated from the wells and washed two times with sterile water. Then, the plate was allowed to air dry for a minimum of 30 minutes. Then, the plate was warmed for 30 minutes in a 37 ^oC non-CO_2 incubator for 30 minutes. Finally, 100 μL of 37 ^oC DMEM/F12 was added to each well of the microplate and the microplate was returned to a 37 ^oC non-CO_2 incubator overnight. The following steps describe actions performed on the day of the assay, in order. XF RPMI was prepared (phenol red-free) (Agilent) was used as the base medium of the assay which was supplemented with 1mM pyruvate (Agilent), 2mM glutamine (Agilent), 11.1 mM glucose (Gibco), and 12.2μM L-Carnitine (Sigma). Using this prepared XF RPMI, drug solutions from the Cell Mito Stress Test kit (Agilent) were resuspended, vortexed for 1 minute, and let rest at room temperature for 1 hour. In this time, the poly-lysine-coated XFe96 spheroid microplates were removed from the incubator and the DMEM/F12 was removed from the plate, washed 1x with 166 μL of prepared XF RPMI, and finally, 175 μL of prepared XF RPMI was added to each well. Then, day 30 organoids in each condition were washed with 166 μL of prepared XF RPMI two times and were transferred to the XFe96 spheroid microplate coated with poly-lysine. Organoids were transferred to the wells using a cut p200 pipette tip. It was ensured that organoids were centered in the well. Following this, the plate was placed in a 37 ^oC non-CO_2 incubator for 1 hour. Drug solutions (Oligomycin, FCCP, and Rot/AA) were loaded into ports A, B, and C, respectively. Port concentrations of oligomycin, FCCP, and Rot/AA were 25μM, 20μM, and 20μM, respectively, such that their final concentrations in solution were 2.5μM, 2μM, and 2μM, respectively. The assay was configured such that the baseline phase ran for 6 cycles, and the oligomycin, FCCP, and Rot/AA stages ran for 10 cycles each. Each cycle constituted a 3-minute mixing, a 0-minute waiting, and a 3-minute measuring phase. Data was normalized to organoid area.
