# Supplementary material for: A patterned human primitive heart organoid model generated by pluripotent stem cell self-organization
Source: Nat Commun. 2023 Dec 12;14:8245. doi: 10.1038/s41467-023-43999-1 (PMC10716495; doi:10.1038/s41467-023-43999-1)
Supplement: Supplementary file 3 — Description of additional supplementary files [file 41467_2023_43999_MOESM3_ESM.pdf]

# Description of Additional Supplementary Files

**Supplementary Data 1.** List of all PCR primers used in this study.

**Supplementary Movie 1** Live imaging of representative Day 30 Control organoid at 10X magnification under brightfield microscopy. Scale bar = 200 µm.

**Supplementary Movie 2** Live imaging of representative Day 30 MM organoid at 10X magnification under brightfield microscopy. Scale bar = 200 µm.

**Supplementary Movie 3** Live imaging of representative Day 30 EMM1 organoid at 10X magnification under brightfield microscopy. Scale bar = 200 µm.

**Supplementary Movie 4** Live imaging of representative Day 30 EMM2/1 organoid at 10X magnification under brightfield microscopy. Scale bar = 200 µm.

**Supplementary Movie 5** Live imaging of calcium transients at 100X magnification within a representative Day 30 Control organoid displays robust beating and calcium activity.

**Supplementary Movie 6** Live imaging of calcium transients at 100X magnification within a representative Day 30 MM organoid displays robust beating and calcium activity.

**Supplementary Movie 7** Live imaging of calcium transients at 100X magnification within a representative Day 30 EMM1 organoid displays robust beating and calcium activity.

**Supplementary Movie 8** Live imaging of calcium transients at 100X magnification within a representative Day 30 EMM2/1 organoid displays robust beating and calcium activity.

**Supplementary Movie 9** 3D reconstruction of confocal immunofluorescence Z-series images of a Day 30 Control organoid stained for DAPI (blue), PECAM1 (green) and TNNT2 (red) showing an internal vascular network. Scale bar = 200 µm.

**Supplementary Movie 10** 3D reconstruction of confocal immunofluorescence Z-series images of a Day 30 MM organoid stained for DAPI (blue), PECAM1 (green) and TNNT2 (red) showing an internal vascular network. Scale bar = 200 µm.

**Supplementary Movie 11** 3D reconstruction of confocal immunofluorescence Z-series images of a Day 30 EMM1 organoid stained for DAPI (blue), PECAM1 (green) and TNNT2 (red) showing an internal vascular network. Scale bar = 200 µm.

**Supplementary Movie 12**
3D reconstruction of confocal immunofluorescence Z-series images of a Day 30 EMM2/1 organoid stained for DAPI (blue), PECAM1 (green) and TNNT2 (red) showing an internal vascular network. Scale bar = 200 µm.

**Supplementary Movie 13**
3D OCT cross-sectional scan of Day 30 Control organoid showing complex, internal chamber morphology and interconnectivity. Scale bar = 400 µm.

**Supplementary Movie 14**
3D OCT cross-sectional scan of Day 30 MM organoid showing complex, internal chamber morphology and interconnectivity. Scale bar = 400 µm.

**Supplementary Movie 15**
3D OCT cross-sectional scan of Day 30 EMM1 organoid showing complex, internal chamber morphology and interconnectivity. Scale bar = 400 µm.

**Supplementary Movie 16**
3D OCT cross-sectional scan of Day 30 EMM2/1 organoid showing complex, internal chamber morphology and interconnectivity. Scale bar = 400 µm.

**Supplementary Movie 17**
Live imaging of representative Untreated day 30 organoid at 20X magnification under brightfield microscopy.

**Supplementary Movie 18**
Live imaging of representative day 30 organoid following 1 µm ondansetron exposure from day 9 to day 20 at 20X magnification under brightfield microscopy.

**Supplementary Movie 19**
Live imaging of representative day 30 organoid following 10 µm ondansetron exposure from day 9 to day 20 at 20X magnification under brightfield microscopy.

**Supplementary Movie 20**
Live imaging of representative day 30 organoid following 100 µm ondansetron exposure from day 9 to day 20 at 20X magnification under brightfield microscopy.
