# Supplementary material for: A patterned human primitive heart organoid model generated by pluripotent stem cell self-organization
Source: Nat Commun. 2023 Dec 12;14:8245. doi: 10.1038/s41467-023-43999-1 (PMC10716495; doi:10.1038/s41467-023-43999-1)
Supplement: Supplementary file 25 — Reporting Summary [file 41467_2023_43999_MOESM25_ESM.pdf]

nature portfolio

Corresponding author(s): Aitor Aguirre

Last updated by author(s): Nov 12, 2023

Reporting Summary

Nature Portfolio wishes to improve the reproducibility of the work that we publish. This form provides structure for consistency and transparency in reporting. For further information on Nature Portfolio policies, see our Editorial Policies and the Editorial Policy Checklist.

Statistics

For all statistical analyses, confirm that the following items are present in the figure legend, table legend, main text, or Methods section.

n/a	Confirmed	
☐	☒	The exact sample size (*n*) for each experimental group/condition, given as a discrete number and unit of measurement
☐	☒	A statement on whether measurements were taken from distinct samples or whether the same sample was measured repeatedly
☐	☒	The statistical test(s) used AND whether they are one- or two-sided<br>*Only common tests should be described solely by name; describe more complex techniques in the Methods section.*
☒	☐	A description of all covariates tested
☐	☒	A description of any assumptions or corrections, such as tests of normality and adjustment for multiple comparisons
☐	☒	A full description of the statistical parameters including central tendency (e.g. means) or other basic estimates (e.g. regression coefficient) AND variation (e.g. standard deviation) or associated estimates of uncertainty (e.g. confidence intervals)
☐	☒	For null hypothesis testing, the test statistic (e.g. *F*, *t*, *r*) with confidence intervals, effect sizes, degrees of freedom and *P* value noted<br>*Give P values as exact values whenever suitable.*
☒	☐	For Bayesian analysis, information on the choice of priors and Markov chain Monte Carlo settings
☒	☐	For hierarchical and complex designs, identification of the appropriate level for tests and full reporting of outcomes
☒	☐	Estimates of effect sizes (e.g. Cohen's *d*, Pearson's *r*), indicating how they were calculated

Our web collection on statistics for biologists contains articles on many of the points above.

Software and code

Policy information about availability of computer code

Data collection	For bioinformatics and data visualization: Microsoft Excel 365 v2210, R.4.3.1, Graphpad Prism 9, Phantasus v1.19.2, Enrichr (https://maayanlab.cloud/Enrichr/).
Data analysis	For statistical analysis and bioinformatics: Microsoft Excel 365 v2210, R.4.3.1, Graphpad Prism 9.<br>For image analysis and visualization: ImageJ 1.50d.<br>For scRNAseq analysis and visualization: R.4.1.3, Phantasus v1.19.2, Enrichr (https://maayanlab.cloud/Enrichr/), Pathview Web (https://pathview.uncc.edu/).

For manuscripts utilizing custom algorithms or software that are central to the research but not yet described in published literature, software must be made available to editors and reviewers. We strongly encourage code deposition in a community repository (e.g. GitHub). See the Nature Portfolio guidelines for submitting code & software for further information.

Data

Policy information about availability of data

All manuscripts must include a data availability statement. This statement should provide the following information, where applicable:

- Accession codes, unique identifiers, or web links for publicly available datasets
- A description of any restrictions on data availability
- For clinical datasets or third party data, please ensure that the statement adheres to our policy

scRNA-Sequencing data sets have been deposited in the National Center for Biotechnology Information Gene Expression Omnibus repository under accession code GSE218582. All data generated and/or analyzed in this study are provided in the published article and its supplementary information files or can be obtained from the corresponding author upon request. Source data are provided with this paper.

Human research participants

Policy information about studies involving human research participants and Sex and Gender in Research.

Reporting on sex and gender	N/A
Population characteristics	N/A
Recruitment	N/A
Ethics oversight	N/A

Note that full information on the approval of the study protocol must also be provided in the manuscript.

Field-specific reporting

Please select the one below that is the best fit for your research. If you are not sure, read the appropriate sections before making your selection.

☒ Life sciences ☐ Behavioural & social sciences ☐ Ecological, evolutionary & environmental sciences

For a reference copy of the document with all sections, see nature.com/documents/nr-reporting-summary-flat.pdf

Life sciences study design

All studies must disclose on these points even when the disclosure is negative.

Sample size	Power analyses of sample size were not done a priori. The rationale for pooling n=4 organoids for scRNAseq was based on a traditional and common resource efficiency approach that also yields robust averaged scRNAseq data and is common across relevant publications ((Kim et. al., 2019, PNAS, PMID: 31072937, Xiang et. al., 2017, Cell Stem Cell, PMID: 28757360). For all other experiments, n=4-14 organoids across three independent experiments were used under most circumstances (unless otherwise stated in the figure legend) where independent experiments are used in accordance with common practice in the field and relevant publications cited in this study (Lee et. al., 2020, Nature Communications, Richards et. al., 2017, Biomaterials, Andersen et. al., 2018, Nature Communications).
Data exclusions	No data were excluded.
Replication	Replications are mentioned in both the figure legends and the Statistics and Reproducibility section of the Methods. Most experiments represent n=4-14 independent organoids per condition across three independent experiments. Technical and experimental replicates performed are noted in figure legends.
Randomization	When multiple organoids were used for experiments, they were always randomly selected according to condition.
Blinding	In the generation and maturation of human heart organoids, the differentiation and application of developmental strategies were performed in a non-blinded manner. Image acquisition was performed with consistent parameters, and threshold measurements were performed with consistent settings and no data was excluded to limit investigator bias.

Reporting for specific materials, systems and methods

We require information from authors about some types of materials, experimental systems and methods used in many studies. Here, indicate whether each material, system or method listed is relevant to your study. If you are not sure if a list item applies to your research, read the appropriate section before selecting a response.

Materials & experimental systems

n/a	Involved in the study
☐	☒ Antibodies
☐	☒ Eukaryotic cell lines
☒	☐ Palaeontology and archaeology
☒	☐ Animals and other organisms
☒	☐ Clinical data
☒	☐ Dual use research of concern

Methods

n/a	Involved in the study
☒	☐ ChIP-seq
☒	☐ Flow cytometry
☒	☐ MRI-based neuroimaging

Antibodies

Antibodies used	TNNT2: Anti-Cardiac Troponin T antibody, (ab8295), Abcam.<br>TNNT2: Anti-Cardiac Troponin T antibody, (ab45932), Abcam.<br>WT1: Recombinant Anti-Wilms Tumor Protein antibody (ab89901), Abcam.<br>PECAM1: Anti-PECAM1 antibody (P2B1), DSHB.<br>MYL2: Anti-Myosin Light Chain 2 antibody (ab79935), Abcam.<br>MYL7: MLC-2A (311-011), Synaptic Systems.<br>TBX18: Anti-TBX18 antibody (ab201587), Abcam.<br>ALDH1A2: Anti-RalDH2 antibody (ABN420), Sigma.<br>KCNJ2: Anti-KCNJ2 antibody (HPA029109), Sigma.<br>NR2F2: Anti-NR2F2 antibody (ab211777), Abcam.<br>MYL3: Anti-MYL3 antibody (Sc-47719), Santa Cruz Biotechnology.<br>Caveolin-3: Anti-caveolin-3 antibody (MAB6706-SP), R&D Systems.<br>Alexa Fluor 488: Donkey anti-Mouse IgG (H+L) Highly Cross-Adsorbed Secondary Antibody, Alexa Fluor 488 (A-21202). ThermoFisher Scientific.<br>Alexa Fluor 488: Donkey anti-Rabbit IgG (H+L) Highly Cross-Adsorbed Secondary Antibody, Alexa Fluor 488 (A-21206). ThermoFisher Scientific.<br>Alexa Fluor 594: Donkey anti-Mouse IgG (H+L) Highly Cross-Adsorbed Secondary Antibody, Alexa Fluor 594 (A-21203). ThermoFisher Scientific.<br>Alexa Fluor 594: Donkey anti-Rabbit IgG (H+L) Highly Cross-Adsorbed Secondary Antibody, Alexa Fluor 594 (A-21207). ThermoFisher Scientific.<br>Alexa Fluor 647: Donkey anti-Rabbit IgG (H+L) Highly Cross-Adsorbed Secondary Antibody, Alexa Fluor 647 (A-31573). ThermoFisher Scientific.
Validation	TNNT2 (ab8295): Suitable for: ICC, IHC-P, Sandwich ELISA. Validated for detecting Troponin T in human cardiac muscle.<br>TNNT2 (ab45932): Suitable for: ICC, IHC-P, Sandwich ELISA. Validated for detecting Troponin T in human cardiac muscle.<br>WT1: Suitable for: WB, IHC-P, Flow Cyt (Intra), ICC/IF. Validated for detecting human Wilms Tumor Protein.<br>PECAM1: Recommended Applications: IF, IHC, IP. Validated for detecting human PECAM1 (CD31).<br>MYL2: Suitable for: WB, IHC-P, IP. Validated for detecting Human Myosin Light Chain 2.<br>MYL7: Suitable for: WB, ICC, IHC, IHC-P/FFPE. Validated for detecting recombinant protein corresponding to AA 1 to 175 from human MLC-2A.<br>TBX18: Suitable for: ICC, WB. Validated for detecting human TBX18.<br>ALDH1A2: Suitable for IF, IHC, WB. Validated for detecting human ALDH1A2.<br>KCNJ2: Suitable for: IHC. Validated for detecting human potassium inwardly-rectifying channel, subfamily J, member 2.<br>NR2F2: Suitable for: WB, IHC-P, ICC/IF, IP. Validated for detecting human NR2F2.<br>MYL3: Suitable for: WB, IP, IF and IHC(P). Validated for detecting human MYL3.<br>Caveolin-3: Suitable for: ICC, ELISA. Validated for detecting human caveolin-3.<br>Alexa Fluor 488: Suitable for: IHC, ICC/IF. Validated for detecting mouse tissue and antibodies.<br>Alexa Fluor 488: Suitable for: IHC, ICC/IF, Flow Cytometry. Validated for detecting rabbit tissue and antibodies.<br>Alexa Fluor 594: Suitable for: IHC, ICC/IF. Validated for detecting mouse tissue and antibodies.<br>Alexa Fluor 594: Suitable for: IHC, IHC(Frozen), ICC/IF, Flow Cytometry. Validated for detecting rabbit tissue and antibodies.<br>Alexa Fluor 647: Suitable for: WB, IHC(Frozen), ICC/IF. Validated for detecting rabbit tissue and antibodies.

Eukaryotic cell lines

Policy information about cell lines and Sex and Gender in Research

Cell line source(s)	Cell line iPSC-L1 was developed in house (iPSC, male, iPSCORE) (Panopoulos et. al., 2017, Stem Cell Reports, PMID: 28410642). Cell line ATCC-BYS0111 was sourced from the American Type Culture Collection (iPSC, male, Cat#: ACS-1025), and the cell line H9 was sourced from WiCell (ESC, female, WiCell, WA09).
Authentication	An array of common iPSC tests were used, including pluripotency testing by using pluripotency marker expression by IF and FACS, and karotyping to confirm genomic integrity and teratoma formation
Mycoplasma contamination	Mycoplasma testing was performed routinely in the lab. All cell lines tested negative.

Commonly misidentified lines<br>(See ICLAC register)	No commonly misidentified cell lines were employed.
